# Rational Recursion Operators for Integrable Differential–Difference Equations

**DOI:** 10.1007/s00220-019-03548-8

**Published:** 2019-08-19

**Authors:** Sylvain Carpentier, Alexander V. Mikhailov, Jing Ping Wang

**Affiliations:** 1 Mathematics Department, Columbia University, New York, USA; 2 School of Mathematics, Statistics and Actuarial Science, University of Kent, Canterbury, UK; 3 Applied Mathematics Department, University of Leeds, Leeds, UK

## Abstract

In this paper we introduce the concept of preHamiltonian pairs of difference operators, demonstrate their connections with Nijenhuis operators and give a criteria for the existence of weakly nonlocal inverse recursion operators for differential–difference equations. We begin with a rigorous setup of the problem in terms of the skew field of *rational* (pseudo–difference) operators over a difference field with a zero characteristic subfield of constants and the principal ideal ring of matrix rational (pseudo–difference) operators. In particular, we give a criteria for a rational operator to be weakly nonlocal. A difference operator is called preHamiltonian, if its image is a Lie subalgebra with respect to the Lie bracket on the difference field. Two preHamiltonian operators form a preHamiltonian pair if any linear combination of them is preHamiltonian. Then we show that a preHamiltonian pair naturally leads to a Nijenhuis operator, and a Nijenhuis operator can be represented in terms of a preHamiltonian pair. This provides a systematic method to check whether a rational operator is Nijenhuis. As an application, we construct a preHamiltonian pair and thus a Nijenhuis recursion operator for the differential–difference equation recently discovered by Adler and Postnikov. The Nijenhuis operator obtained is not weakly nonlocal. We prove that it generates an infinite hierarchy of local commuting symmetries. We also illustrate our theory on the well known examples including the Toda, the Ablowitz–Ladik, and the Kaup–Newell differential–difference equations.

## Introduction

The existence of an infinite hierarchy of commuting symmetries is one of a characteristic property of integrable systems. Symmetries can be generated by recursion operators [1, 2], which are often pseudo–differential and map a symmetry to a new symmetry. An important property of recursion operators, called the Nijenhuis property, is to generate an abelian Lie algebra of symmetries. Such property has been independently studied by Fuchssteiner [3] and Magri [4]. To prove that a pseudo–differential operator is a Nijenhuis operator and it generates an infinite hierarchy of local symmetries is a challenging problem. In the most common case of weakly nonlocal Nijenhuis operators this problem has been addressed in [5–7]. The relations between bi-Hamiltonian structures and Nijenhuis operators have been studied in papers of Gel’fand and Dorfman [8, 9] and Fuchssteiner and Fokas [10, 11]. Recently a rigorous approach to pseudo–differential Hamiltonian operators have been developed in the series of papers by Barakat, De Sole, Kac and Valeri [12–14].

The theory of integrable differential–difference equations is much less developed. The basic concepts for symmetries, conservation laws and Hamiltonian operators were formulated in the frame of a variational complex in [15]. The aim of this paper is to build up a rigorous setting for rational matrix (pseudo–difference) operators suitable for the study of integrable differential–difference systems. We introduce and study preHamiltonian pairs of difference operators, their connections with Nijenhuis operators and the existence of weakly nonlocal inverse recursion operators for differential–difference equations.

Let us consider the well-known Volterra chain

1 $$\begin{aligned} u_t=u (u_1-u_{-1}) , \end{aligned}$$

where *u* is a function of a lattice variable $n\in {\mathbb {Z}}$ and time *t*. Here we use the notations

$$\begin{aligned} u_t=\partial _t(u), \quad u_j={\mathcal S}^j u(n,t)=u(n+j,t) \end{aligned}$$

and ${\mathcal S}$ is the shift operator. It possesses a recursion operator

$$\begin{aligned} R=u {\mathcal S}+u +u_1+u {\mathcal S}^{-1}+u(u_1-u_{-1}) ({\mathcal S}-1)^{-1}\frac{1}{u} , \end{aligned}$$

where $({\mathcal S}-1)^{-1}$ stands for the inverse of ${\mathcal S}-1$. Thus this operator is only defined on $u{\text {Im}}({\mathcal S}-1)$. It is a Nijenhuis operator and generates a commutative hierarchy of symmetries:

$$\begin{aligned} u_{t_j}=R^j (u_t)=R^j\left( u(u_1-u_{-1})\right) ,\qquad j=0, 1, 2,\ldots . \end{aligned}$$

The concept of Hamiltonian pairs was introduced by Magri [16]. He found that some systems admitted two distinct but compatible Hamiltonian structures (a Hamiltonian pair) and named them twofold Hamiltonian system, nowadays known as bi-Hamiltonian systems. The Volterra chain is a bi-Hamiltonian system and it can be written

$$\begin{aligned} u_t=H_1\ \delta _u u=H_2\ \delta _u \frac{\ln u}{2}, \end{aligned}$$

where $\delta _u$ is variational derivative with respect to the dependent variable *u* and two difference operators

$$\begin{aligned}&H_1=u({\mathcal S}-{\mathcal S}^{-1})u;\\&H_2=u({\mathcal S}u{\mathcal S}+u {\mathcal S}+{\mathcal S}u-u {\mathcal S}^{-1}-{\mathcal S}^{-1} u-{\mathcal S}^{-1} u{\mathcal S}^{-1})u \end{aligned}$$

form a Hamiltonian pair. The Nijenhuis recursion operator of the Volterra chain can be obtained via the Hamiltonian pair, that is, $$ R=H_2 H_1^{-1}$$. This decomposition is known as the Lenard scheme used to construct the hierarchies of infinitely many symmetries and cosymmetries.

Notice that the above difference operators have a right common factor:

$$\begin{aligned}&H_1=u ({\mathcal S}-1)(1+{\mathcal S}^{-1})u;\qquad H_2 =u(1+{\mathcal S})(u {\mathcal S}-{\mathcal S}^{-1} u) (1+{\mathcal S}^{-1}) u . \end{aligned}$$

This implies that

2 $$\begin{aligned} R=A B^{-1}, \ \text{ where } A=u({\mathcal S}+1)(u {\mathcal S}-{\mathcal S}^{-1} u)\hbox { and }B=u ({\mathcal S}-1). \end{aligned}$$

Here operators *A* and *B* are not skew-symmetric, and thus not Hamiltonian. However, like in the case of Hamiltonian pairs, the image of *A* and *B*, as well as the image of linear combinations of these two operators, form a Lie subalgebra. Such operators we call preHamiltonian operator. In this paper, we explore properties of such operators and their relations with Nijenhuis operators. For the differential case some of these results have been obtained in [17]. The main difference between differential operators and difference operators lies in that the total derivative is a derivation and the shift operator ${\mathcal S}$ is an automorphism. The set of invertible difference operators is much richer than in the differential case. In the scalar case all difference operators of the form $a {\mathcal S}^j$, where *a* is a difference function and $j\in \mathbb {Z}$, are invertible, while in the differential case, the only invertible operators are operators of multiplication by a function. The definition of the order of difference and differential operators are essentially different.

The arrangement of this paper is as follows: In Sect. 2, we define a difference field ${\mathrm F}$, the Lie algebra ${\mathcal {A}}$ of its evolutionary derivations (or evolutionary vector fields) which is a subalgebra of $\mathrm{Der}\,{\mathrm F}$ and discuss algebraic properties of the noncommutative ring of difference operators. In particular, we show that it is a right and left Euclidean domain and satisfies the right (left) Ore property. Then we define the skew field of rational (pseudo–difference) operators, i.e. operators of the form $AB^{-1}$, where *A* and *B* are difference operators. Next we discuss the relation between rational operators and weakly nonlocal operators, namely we formulate a criteria for a rational operator to be weakly nonlocal. Finally we adapt all these results to rational matrix difference operators by defining the order of the operator as the order of its Dieudonné determinant. In Sect. 3 we define preHamiltonian difference operators as operators on ${\mathrm F}$ whose images define a Lie subalgebra in ${\mathcal {A}}$. We explore the interrelation between preHamiltonian pairs and Nijenhuis operators. We show that if operators *A* and *B* form a preHamiltonian pair, then $R=AB^{-1}$ is Nijenhuis. Conversely, if *R* is Nijenhuis and *B* is preHamiltonian, then *A* and *B* form a preHamiltonian pair. These two sections are the theoretical foundation of the paper. In Sect. 4, we give basic definitions such as symmetries, recursion operators and Hamiltonian for differential–difference equations. We also show how operators *A* and *B* are related to the equation if $AB^{-1}$ is its recursion operator. In the next two sections we apply the theoretical results in Sects. 2 and 3 to integrable differential–difference equations. In Sect. 5, we construct a recursion operator for a new integrable equation derived by Adler and Postnikov in [18]:

$$\begin{aligned} u_t=u^2(u_2u_1-u_{-1}u_{-2})-u(u_1-u_{-1}), \end{aligned}$$

using its Lax representation presented in the same paper. The obtained recursion operator is no longer weakly nonlocal. We show that it is indeed Nijenhuis by rewriting it as a rational difference operator and that it generates infinitely many commuting local symmetries. To improve the readability, we put some technical lemmas used for the proof of the main result on the locality of commuting symmetries in “Appendix B”. For some integrable differential–difference equations, such as the Ablowitz–Ladik Lattice [19], the recursion operator and its inverse are both weakly nonlocal. In Sect. 6, we apply the theoretical results from Sect. 2 to check whether the inverse recursion operators are weakly nonlocal, and if so, we demonstrate how to cast them in the weakly nonlocal form. To illustrate the method we choose four typical examples. However, the method is general and it can be applied to any integrable differential–difference system, including all systems listed in [20]. At the end of the paper we give a short conclusion and discussion on our new results on relation between preHamiltonian and Hamiltonian operators. To be self-contained, we also include “Appendix A”, containing some basic definitions for a unital non-commutative ring.

## Algebraic Properties of Difference Operators

In this section, we give a definition of rational difference operators and explore their properties. The main objects of our study in this paper are systems of evolutionary differential–difference equations and hidden structures associated with them. We first consider the scalar case. A generalization to the multi-component case will be discussed in the end of this section.

### Difference field and its derivations

Let ${\mathrm k}$ be a zero characteristic base field, such as ${\mathbb {C}}$ or ${\mathbb {R}}$. We define the polynomial ring

$$\begin{aligned} \mathrm {K}={\mathrm k}[\ldots , u _{-1}, u _0, u _1,\ldots ] \end{aligned}$$

of the infinite set of variables $\{u\}=\{ u _k;\ k\in {\mathbb {Z}}\}$ and the corresponding field of fractions

$$\begin{aligned} {\mathrm F}={\mathrm k}(\ldots , u _{-1}, u _0, u _1,\ldots ). \end{aligned}$$

It is assumed that every element of $\mathrm {K}$ and ${\mathrm F}$ depends on a finite number of variables only. We will denote ${\mathrm F}^\star$ the subset of nonzero elements ${\mathrm F}^\star ={\mathrm F}{\setminus }\{0\}$ of ${\mathrm F}$.

There is a natural automorphism ${\mathcal S}$ of the field ${\mathrm F}$, which we call the shift operator, defined as

$$\begin{aligned} {\mathcal S}: a( u _k,\ldots , u _r)\mapsto a( u _{k+1},\ldots , u _{r+1}),\quad {\mathcal S}:\alpha \mapsto \alpha , \qquad a( u _k,\ldots , u _r)\in {\mathrm F},\ \ \alpha \in {\mathrm k}. \end{aligned}$$

For $a=a( u _k,\ldots , u _r)\in {\mathrm F}$ we will often use notation

$$\begin{aligned} a_i={\mathcal S}^i(a)=a( u _{k+i},\ldots , u _{r+i}),\qquad i\in {\mathbb {Z}}, \end{aligned}$$

and omit index zero at $a_0$ or $u_0$ when there is no ambiguity. The field ${\mathrm F}$ equipped with the automorphism ${\mathcal S}$ is a difference field and the base field ${\mathrm k}$ is its subfield of constants.

The reflection ${\mathcal {T}}$ of the lattice ${\mathbb {Z}}$ defined by

$$\begin{aligned} {\mathcal {T}}: a( u _k,\ldots , u _r)\mapsto a(u_{-k},\ldots ,u_{-r}),\quad {\mathcal {T}}:\alpha \mapsto \alpha , \qquad a( u _k,\ldots , u _r)\in {\mathrm F},\ \ \alpha \in {\mathrm k}, \end{aligned}$$

is another obvious automorphism of ${\mathrm F}$ and $\mathrm {K}$. The composition ${\mathcal S}{\mathcal {T}}{\mathcal S}{\mathcal {T}}=\text{ Id }$ is the identity map. Thus the automorphisms ${\mathcal S},{\mathcal {T}}$ generate the infinite dihedral group ${\mathbb {D}}_\infty$ and the subgroup generated by ${\mathcal S}$ is normal.

The automorphism ${\mathcal {T}}$ defines a ${\mathbb {Z}}_2$ grading of the difference field ${\mathrm F}$ (and ring $\mathrm {K}\subset {\mathrm F}$):

$$\begin{aligned} {\mathrm F}={\mathrm F}_0\oplus {\mathrm F}_1,\qquad {\mathrm F}_0\cdot {\mathrm F}_0={\mathrm F}_0,\ \ {\mathrm F}_0\cdot {\mathrm F}_1={\mathrm F}_1,\ \ {\mathrm F}_1\cdot {\mathrm F}_1={\mathrm F}_0, \end{aligned}$$

where $$ {\mathrm F}_k=\{a\in {\mathrm F}\,|\, {\mathcal {T}}(a)=(-1)^k a\}$$.

Partial derivatives $\frac{\partial }{\partial u_i},\ i\in {\mathbb {Z}}$ are commuting derivations of ${\mathrm F}$ satisfying the conditions

3 $$\begin{aligned} {\mathcal S}\frac{\partial }{\partial u_{i}}= \frac{\partial }{\partial u_{i+1}} {\mathcal S}, \qquad {\mathcal {T}}\frac{\partial }{\partial u_{i}}= \frac{\partial }{\partial u_{-i}} {\mathcal {T}}. \end{aligned}$$

A derivation of ${\mathrm F}$ is said to be evolutionary if it commutes with the shift operator ${\mathcal S}$. Such derivation is completely determined by one element of $f\in {\mathrm F}$ and is of the form

4 $$\begin{aligned} X_f=\sum _{i\in {\mathbb {Z}}}{\mathcal S}^i(f) \frac{\partial }{\partial u_{i}},\qquad f\in {\mathrm F}. \end{aligned}$$

An element *f* is called the characteristic of the evolutionary derivation $X_f$. The action of $X_f(a)$ for $a\in {\mathrm F}$ can also be represented in the form

$$\begin{aligned} X_f(a)=a_*[f], \end{aligned}$$

where $a_*[f]$ is the Fréchet derivative of $a=a(u_p,\ldots ,u_q)$ in the direction *f* defined as

$$\begin{aligned} a_*[f]:= \frac{d}{d\epsilon }a(u_p+\epsilon f_p,\ldots ,u_q+\epsilon f_q)\arrowvert _{\epsilon =0}=\sum _{i=p}^q\frac{\partial a}{\partial u_i}f_i. \end{aligned}$$

The Fréchet derivative of $a=a(u_p,\ldots ,u_q)$ is a difference operator represented by a finite sum

5 $$\begin{aligned} a_*=\sum _{i=p}^q\frac{\partial a}{\partial u_i}{\mathcal S}^i. \end{aligned}$$

It is obvious that

$$\begin{aligned} ({\mathcal {T}}a)_*=\sum _{i=p}^q{\mathcal {T}}\left( \frac{\partial a}{\partial u_i}\right) {\mathcal S}^{-i}. \end{aligned}$$

Evolutionary derivations form a Lie subalgebra ${\mathcal {A}}$ in the the Lie algebra $\mathrm{Der}\, {\mathrm F}$. Indeed,

$$\begin{aligned}\begin{array}{l} \alpha X_f+\beta X_g=X_{\alpha f+\beta g},\qquad \alpha ,\beta \in {\mathrm k},\\ \phantom {} [X_f,X_g]=X_{[f,g]}, \end{array} \end{aligned}$$

where $[f,g]\in {\mathrm F}$ denotes the Lie bracket

6 $$\begin{aligned}{}[f,g]=X_f(g)-X_g(f)=g_*[f]-f_*[g]. \end{aligned}$$

Lie bracket (6) is ${\mathrm k}$–bilinear, anti-symmetric and satisfies the Jacobi identity. Thus ${\mathrm F}$, equipped with the bracket (6), has a structure of a Lie algebra over ${\mathrm k}$.

The reflection ${\mathcal {T}}$ acts naturally on evolutionary vector derivations

$$\begin{aligned} {\mathcal {T}}:\, X_f\mapsto X_{{\mathcal {T}}(f)}={\mathcal {T}}\cdot X_f\cdot {\mathcal {T}}. \end{aligned}$$

Thus the ${\mathcal {A}}$ is a graded Lie algebra

$$\begin{aligned} {\mathcal {A}}={\mathcal {A}}_0\oplus {\mathcal {A}}_1,\qquad [{\mathcal {A}}_0,{\mathcal {A}}_0]\subset {\mathcal {A}}_0,\ [{\mathcal {A}}_0,{\mathcal {A}}_1]\subset {\mathcal {A}}_1,\ [{\mathcal {A}}_1,{\mathcal {A}}_1]\subset {\mathcal {A}}_0,\ \end{aligned}$$

where $${\mathcal {A}}_k=\{X\in {\mathcal {A}}\,|\, {\mathcal {T}}(X)=(-1)^k X\}$$.

### Rational difference operators

In this section we give definitions of difference operators and rational pseudo–difference operators, which for simplicity we shall call rational operators. We refer to the “Appendix A” for general results and definitions related to principal ideal domains. Although Corollary 1 and the first part of Proposition 2 follow directly from Proposition 1 in the abstract setting of Euclidean domains, we provide complete proofs for the sake of completeness.

#### Definition 1

A difference operator *B* of order $\mathrm{ord}\, B:=(M,N)$ with coefficients in ${\mathrm F}$ is a finite sum of the form

7 $$\begin{aligned} B= & {} {} b^{(N)} {\mathcal S}^{N}+b^{(N-1)} {\mathcal S}^{N-1}+\cdots +b^{(M)} {\mathcal S}^{M},\quad b^{(N)} b^{(M)}\ne 0,\ b^{(k)}\in {\mathrm F}, \ \nonumber \\&M\le N, \ N,M\in \mathbb {Z}. \end{aligned}$$

The total order of *B* is defined as $\mathrm{Ord}B=N-M$. The total order of the zero operator is minus infinity $\mathrm{Ord}\, 0:=-\infty$ by definition.

The Fréchet derivative (5) is an example of a difference operator of order (*p*, *q*) and total order $\mathrm{Ord}\, a_* =q-p$. For an element $f\in {\mathrm F}$ the order and total order are defined as $\mathrm{ord}\, f_*$ and $\mathrm{Ord}\, f_*$ respectively.

Difference operators form a unital ring ${\mathcal {R}}={\mathrm F}[{\mathcal S},{\mathcal S}^{-1}]$ of Laurent polynomials in ${\mathcal S}$ with coefficients in ${\mathrm F}$, where multiplication is defined by

8 $$\begin{aligned} a{\mathcal S}^n \cdot b{\mathcal S}^m=a{\mathcal S}^n(b){\mathcal S}^{n+m}=a b_n {\mathcal S}^{n+m}. \end{aligned}$$

This multiplication is associative, but non-commutative. The definitions of some basic concepts for a unital associative ring are presented in the “Appendix A”.

From the above definition it follows that if *A* is a difference operator of order $\mathrm{ord}\, A=(p,q)$, then $\mathrm{ord}\, ({\mathcal S}^n\cdot A\cdot {\mathcal S}^m) =(p+n+m,q+n+m)$ and $\mathrm{Ord}\, ({\mathcal S}^n\cdot A\cdot {\mathcal S}^m )=\mathrm{Ord}\, A=q-p$. For any $A,B\in {\mathcal {R}}$ we have $\mathrm{Ord}\, (AB)=\mathrm{Ord}\, A+\mathrm{Ord}\, B$. Thus the total order is homomorphisms of the multiplicative monoid ${\mathcal {R}}$ to ${\mathbb {Z}}_{\ge 0}\cup \{\infty \}$.

The reflection ${\mathcal {T}}$ can be extended to an automorphism of ${\mathcal {R}}$ given by

$$\begin{aligned} {\mathcal {T}}\cdot a{\mathcal S}^m\cdot {\mathcal {T}}={\mathcal {T}}(a){\mathcal S}^{-m} \end{aligned}$$

and defines a grading of ${\mathcal {R}}$ as follows:

$$\begin{aligned} {\mathcal {R}}={\mathcal {R}}_0\oplus {\mathcal {R}}_1,\qquad {\mathcal {R}}_k=\{A\in {\mathcal {R}}\,|\, {\mathcal {T}}\cdot A\cdot {\mathcal {T}}=(-1)^k A\}. \end{aligned}$$

It is obvious that $\mathrm{Ord}({\mathcal {T}}\cdot A\cdot {\mathcal {T}})=\mathrm{Ord}\, A$.

A difference operator which has only one term $a{\mathcal S}^{n},\ a\in {\mathrm F}^*,\ n\in {\mathbb {Z}}$ is called a *monomial* difference operator. The set of monomial difference operators are of the form $a{\mathcal S}^n,\ a\ne 0$. They have total order equal to zero and are invertible in ${\mathcal {R}}$. Monomial difference operators equipped with multiplication (8) form a nonabelian group

$$\begin{aligned} {\mathcal {R}}^\star =\{a{\mathcal S}^n\, |\, a\in {\mathrm F}^\star ,\ n\in {\mathbb {Z}}\}. \end{aligned}$$

We will use the notation $\text{ LT }(B)$ for a monomial difference operator representing the leading term of a difference operator which is the naturally ordered sum. For the operator *B* in (7), we have $\text{ LT }(B)=b^{(N)} {\mathcal S}^{N}$.

#### Proposition 1

The ring ${\mathcal {R}}$ is a right and left Euclidean domain.

#### Proof

Let us show that ${\mathcal {R}}$ is a right Euclidean, that is, for any $A,B\in {\mathcal {R}}$ there exist unique $Q, R\in {\mathcal {R}}$ such that $A=B\cdot Q+R$ and either $R=0$ or $\mathrm{Ord}\, R<\mathrm{Ord}\, B$. First we prove the existence of *Q*, *R*. If $A=0$, then we can take $Q=R=0$. If $A\ne 0$ and $\mathrm{Ord}\, A<\mathrm{Ord}\, B$, we can take $Q=0,\ R=A$. For $\mathrm{Ord}\, A\ge \mathrm{Ord}\, B$ we proceed by induction on $\mathrm {Ord}\, A=0\ (=\mathrm {Ord}\, B)$, then $A=a{\mathcal S}^N,\ B=b{\mathcal S}^M$ for some $N,M\in {\mathbb {Z}}$ and they are invertible. Thus $A=BB^{-1}A$ and we can take $R=0, \ Q=B^{-1}A={\mathcal S}^{-M}(a/b){\mathcal S}^{N-M}$. Finally, consider the case $\mathrm{Ord}\, A=n\ge 1,\ \mathrm{Ord}\, B=m,\ n\ge m$ and assume that the statement is true for all operators *A* with total order less than *n*. Let the leading terms $\text{ LT }(A)= a{\mathcal S}^N$ and $\text{ LT }(B)=b{\mathcal S}^M$. The difference operator ${\hat{A}}=A-B \cdot (b{\mathcal S}^M)^{-1}\cdot a{\mathcal S}^N$ has $\mathrm{Ord}\, {\hat{A}}<\mathrm{Ord}\, A=n$. Hence we can use the induction assumption and find $\hat{Q},\ {\hat{R}}$, such that ${\hat{A}}=B{\hat{Q}}+{\hat{R}}$ and either ${\hat{R}}=0$ or $\mathrm{Ord}\, \hat{R}<\mathrm{Ord}B$. Thus

$$\begin{aligned} A-B \cdot (b{\mathcal S}^M)^{-1}\cdot a{\mathcal S}^N=B{\hat{Q}}+{\hat{R}}, \end{aligned}$$

that is,

$$\begin{aligned} A=B((b{\mathcal S}^M)^{-1}\cdot a{\mathcal S}^N+{\hat{Q}})+{\hat{R}}. \end{aligned}$$

Therefore $Q=(b{\mathcal S}^M)^{-1}\cdot a{\mathcal S}^N+{\hat{Q}}$ and $R={\hat{R}}$. As for the uniqueness, if one has $BQ+R=B{\tilde{Q}}+{\tilde{R}}$ with $\mathrm{Ord}\, R<\mathrm{Ord}B,\ \mathrm{Ord}\, {\tilde{R}}<\mathrm{Ord}B$, then $B(Q-{\tilde{Q}})={\tilde{R}}-R$. If $Q\ne {\tilde{Q}}$ we arrive to a contradiction since $\mathrm{Ord}(B(Q-{\tilde{Q}}))>\mathrm{Ord}({\tilde{R}}-R)$. Thus $Q= {\tilde{Q}}$ and $R= {\tilde{R}}$. The proof of the left Euclidean property is similar. $\quad \square$

#### Corollary 1

Every right (left) ideal of the ring ${\mathcal {R}}$ is principal and generated by a unique element $A\in {\mathcal {R}}$ of minimal possible order with the leading term $\mathrm{LT}(A)=1$.

#### Proof

The zero ideal is obviously principal, it is generated by 0. Let $J\subset {\mathcal {R}}$ be a right ideal and ${\hat{A}}\in J$ be an element of least possible total order. The element $A={\hat{A}}\cdot \mathrm{LT}(\hat{A})^{-1}\in J$, is of the same total order and with the leading term $\mathrm{LT}(A)=1$. Then for any other element $B\in J$ we have $B=AQ+R$ with either $R=0$ or $\mathrm{Ord}\, R<\mathrm{Ord}\, A$. Since $B\in J$, we conclude that $R=0$, otherwise $\mathrm{Ord}\, R<\mathrm{Ord}\, A$, which is in contradiction with the assumption that *A* has the least possible order. Such element *A* is obviously unique. If we assume the existence of ${\tilde{A}}\in J, \ \mathrm{Ord}\, {\tilde{A}}=\mathrm{Ord}\, A,\ \mathrm{LT}({\tilde{A}})=1$, then $A-{\tilde{A}}\in J$ and $\mathrm{Ord}\, (A-{\tilde{A}})<\mathrm{Ord}\, A$. The latter is in contradiction with the assumption that *A* has the least possible order. In a similar way we show that ${\mathcal {R}}$ is a left principal ideal ring. $\quad \square$

#### Proposition 2

The ring ${\mathcal {R}}$ satisfies the right (left) Ore property, that is, for any $A,B\in {\mathcal {R}}$ their exist $A_1,B_1$, not both equal to zero, such that $AB_1=BA_1$, (resp. $B_1 A=A_1 B$). In other words, the right (left) ideal $A {\mathcal {R}}\cap B {\mathcal {R}}$ (resp. ${\mathcal {R}}A \cap {\mathcal {R}}B$) is nontrivial. Its generator *M* has total order $\mathrm{Ord}A+ \mathrm{Ord}B-\mathrm{Ord}D$, where *D* is the greatest left (resp. right) common divisor of *A* and *B*.

#### Proof

Let us assume that $\mathrm{Ord}\, A\ge \mathrm{Ord}\, B$ (otherwise we swap and rename *A*, *B*). If $B=0$, then $B_1=0$. If $B\ne 0$, we prove the claim by induction on $\mathrm{Ord}\, B$. We assume that the statement is true for any *B* with $\mathrm{Ord}\, B<k$ and we will show that it is also true for any $B,\ \mathrm{Ord}\,B=k$. Since ${\mathcal {R}}$ is right Euclidean, there exist *Q*, *R* such that $A=BQ+R$ and either $R=0$ or $\mathrm{Ord}\, R<\mathrm{Ord}B$. If $R=0$ we take $A_1=Q,\ B_1=1$ and we are done. Since $\mathrm{Ord}\, R<k$, there exist ${\hat{B}}$, ${\hat{R}}$ such that $B {\hat{R}}=R {\hat{B}}$, $\mathrm{Ord}{\hat{R}} \le \mathrm{Ord}R$ and $\mathrm{Ord}{\hat{B}} \le \mathrm{Ord}B$. Thus

$$\begin{aligned} A {\hat{B}}=(BQ+R){\hat{B}}\ \Leftrightarrow \ A {\hat{B}}=B (Q {\hat{B}}+{\hat{R}}) \end{aligned}$$

and we can take $A_1=Q{\hat{B}}+ {\hat{R}}$, $B_1={\hat{B}}$. Finally, we can see that $\mathrm{Ord}A_1 \le \mathrm{Ord}A$ and $\mathrm{Ord}B_1 \le \mathrm{Ord}B$. The proof of the left Ore property is similar.

We proved that for any $A, B \in {\mathcal {R}}$ not both zero, the ideal $\mathcal {I}=A {\mathcal {R}}\cap B {\mathcal {R}}$ is not trivial. Since ${\mathcal {R}}$ is both a right and left principal ideal ring, $\mathcal {I}$ is generated by a difference operator *M*, $\mathcal {I}=M {\mathcal {R}}$. In particular, $M=AB_1=BA_1$ for some difference operators $B_1$ and $A_1$. From the first part of the proof, we know that $\mathrm{Ord}M \le \mathrm{Ord}A+ \mathrm{Ord}B$. Let us assume that *A* and *B* are left coprime and that $\mathrm{Ord}M< \mathrm{Ord}A+\mathrm{Ord}B$. The ideal $\mathcal {J}={\mathcal {R}}A_1 \cap {\mathcal {R}}B_1$ is also nontrivial and generated by a difference operator *N*. We know that $\mathrm{Ord}N$ is at most $\mathrm{Ord}A_1 + \mathrm{Ord}B_1$. *M* is an element of $\mathcal {J}$ and $\mathrm{Ord}M=\mathrm{Ord}A + \mathrm{Ord}B_1> \mathrm{Ord}A_1+ \mathrm{Ord}B_1 \ge \mathrm{Ord}N$, hence there exists a difference operator *C* such that $M=CN$ and $\mathrm{Ord}C >0$. Let $A_2$ and $B_2$ be such that $A_2B_1=B_2A_1=N$. Then $A=CA_2$ and $B=CB_2$, which contradicts the hypothesis that *A* and *B* are left coprime. $\quad \square$

The fact that ${\mathcal {R}}$ is a principal ideal domain gives sense to the notions of greatest common divisors and least common multiples (see “Appendix A”). The following lemma, which will be used in Proposition 13, relates the images of two difference operators to the image of their right least common multiple.

#### Lemma 1

Let *A* and *B* be two nonzero left coprime difference operators with coefficients in ${\mathrm F}$. Suppose that $A(x)=B(y)$ for some $x,y \in {\mathrm F}$. Let $M=AC=BD$ be their right least common multiple. Then, there exists $z \in {\mathrm F}$ such that $x=C(z)$ and $y=D(z)$. In particular $Im A \cap Im B =Im M$.

#### Proof

By definition of *M*, *C* and *D* are right coprime. It follows from the Bezout’s Lemma that there exist two difference operators *U* and *V* such that

9 $$\begin{aligned} UC+VD=1. \end{aligned}$$

Multiplying (9) on *D* and on *C* from the left we obtain

10 $$\begin{aligned}&DUC=(1-DV)D, \end{aligned}$$

11 $$\begin{aligned}&CVD=(1-CU)C. \end{aligned}$$

By assumption *A* and *B* are left coprime therefore it follows from Lemma 5 (ii) that there exist two difference operators *P* and *Q* such that

12 $$\begin{aligned} \begin{aligned}&1-DV=PB, \quad DU=PA\\&1-CU=QA, \quad CV=QB. \end{aligned} \end{aligned}$$

Using the assumption $A(x)=B(y)$ and the first line of (12) we get

13 $$\begin{aligned} y=(PB+DV)(y)=PA(x)+DV(y)=D(U(x)+V(y)), \end{aligned}$$

and similarly using the second line of (12) we get

14 $$\begin{aligned} x=(CU+QA)(x)=CU(x)+QB(x)=C(U(x)+V(y)). \end{aligned}$$

Hence, the statement holds with $z=U(x)+V(y)$. $\quad \square$

The domain ${\mathcal {R}}$ can be naturally embedded in the skew field of rational pseudo–difference operators, which we will call simply *rational operators*.

#### Definition 2

A rational (pseudo–difference) operator *L* is defined as $L=AB^{-1}$ for some $A,B\in {\mathcal {R}}$ and $B\ne 0$. The set of all rational operators is

$$\begin{aligned} {\mathcal {Q}}=\{ AB^{-1}\,|\, A,B\in {\mathcal {R}},\ B\ne 0\}. \end{aligned}$$

#### Remark 1

The skew field ${\mathcal {Q}}$ is a minimal subfield of the skew field ${\mathcal {Q}}^L$ of the Laurent formal series

$$\begin{aligned} {\mathcal {Q}}^L=\left\{ \sum _{n=k}^\infty a^{(-n)}{\mathcal S}^{-n}\ |\ a^{(l)}\in {\mathrm F},\ l\in {\mathbb {Z}}\right\} \end{aligned}$$

containing ${\mathcal {R}}$. As well as it is a minimal subfield of the skew field ${\mathcal {Q}}^T$ of the Taylor formal series

$$\begin{aligned} {\mathcal {Q}}^T=\left\{ \sum _{n=k}^\infty a^{(n)}{\mathcal S}^{n}\ |\ a^{(l)}\in {\mathrm F},\ l\in {\mathbb {Z}}\right\} \end{aligned}$$

containing ${\mathcal {R}}$. The skewfields ${\mathcal {Q}}^L$ and ${\mathcal {Q}}^T$ are isomorphic. The isomorphism is given by the reflection map ${\mathcal {T}}$.

#### Proposition 3

Any rational operator $L=AB^{-1}$ can also be written in the form $L=\hat{B}^{-1}{\hat{A}}$ with ${\hat{A}},\hat{B}\in {\mathcal {R}}$ and ${\hat{B}}\ne 0$.

#### Proof

It follows from the Ore property that for any $A,B\in {\mathcal {R}},\ B\ne 0$ there exist $\hat{A},\hat{B}\in {\mathcal {R}}$ and ${\hat{B}}\ne 0$ such that $\hat{B}A=\hat{A}B$. Multiplying this expression on ${\hat{B}}$ from the left and $B^{-1}$ from the right we obtain $L=AB^{-1}=\hat{B}^{-1}{\hat{A}}$. $\quad \square$

Thus any statement for the representation $L=AB^{-1}$ can be easily reformulated to the representation $L={\hat{B}}^{-1}{\hat{A}}$. In particular,

$$\begin{aligned} {\mathcal {Q}}=\{ AB^{-1}\,|\, A,B\in {\mathcal {R}},\ B\ne 0\}=\{ B^{-1}A\,|\, A,B\in {\mathcal {R}},\ B\ne 0\}. \end{aligned}$$

#### Proposition 4

${\mathcal {Q}}$ is the skew field of rational operators over ${\mathrm F}$.

#### Proof

We need to show that the set ${\mathcal {Q}}$ is closed under addition and multiplication. Let $A,B,C,D\in {\mathcal {R}}$ with $B\ne 0, D\ne 0$. It follows from the Ore property that there exist nonzero ${\hat{B}},{\hat{D}}\in {\mathcal {R}}$ such that $B{\hat{D}}=D{\hat{B}}$. Hence

$$\begin{aligned} AB^{-1}+CD^{-1}=(A{\hat{D}}+C{\hat{B}})\cdot (B{\hat{D}})^{-1}\in {\mathcal {Q}}. \end{aligned}$$

Also there exist nonzero ${\hat{B}},{\hat{C}}$ such that $B{\hat{C}}=C{\hat{B}}$. Hence

$$\begin{aligned} (AB^{-1})\cdot (CD^{-1})=(A{\hat{C}})\cdot (D{\hat{B}})^{-1} \in {\mathcal {Q}}\end{aligned}$$

implying that ${\mathcal {Q}}$ is also closed under multiplication. $\quad \square$

#### Proposition 5

The decomposition $L=AB^{-1},\ A,B\in {\mathcal {R}}$ of an element $L\in {\mathcal {Q}}$ is unique if we require that *B* has a minimal possible total order with leading term $\mathrm{LT}(B)=1$. For any other decomposition $L=\hat{A}{\hat{B}}^{-1},\ {\hat{A}},{\hat{B}}\in {\mathcal {R}}$ there exists $C\in {\mathcal {R}}$ such that ${\hat{A}}=AC,\ {\hat{B}}=BC$. Moreover, if $D^{-1}E$ is a (left) minimal decomposition of *L*, then $\mathrm{Ord}D=\mathrm{Ord}B$.

#### Proof

For a given $L\in {\mathcal {Q}}$ the set

$$\begin{aligned} J=\{X \in {\mathcal {R}}\, |\, LX \in {\mathcal {R}}\} \end{aligned}$$

is a right ideal in ${\mathcal {R}}$. Indeed, if $X, Y \in J$, then $L(X+Y)=LX+LY \in {\mathcal {R}}$ meaning that $X+Y \in J$, and *J* is stable under right multiplication by any element of ${\mathcal {R}}$. The ideal *J* is principal, and according to Corollary 1 it is generated by a unique element *B* of the least possible order, if we require that the leading term $\mathrm{LT}(B)=1$. Any other ${\hat{B}}\in J$ can be represented as ${\hat{B}}=BC$ where $C\in {\mathcal {R}}$, since *B* is a generator of the principal right ideal *J*. By Proposition 2, we know that a generator *M* of the left ideal generated by *A* and *B* has total order $\mathrm{Ord}A+ \mathrm{Ord}B$. By definition of *M* there exist left coprime difference operators *D* and *E* such that $DA=EB=M$. Therefore $D^{-1}E$ is a left minimal decomposition of *L* and $\mathrm{Ord}D=\mathrm{Ord}B$. $\quad \square$

The definition of total order for difference operators (Definition 1) can be extended to rational operators:

15 $$\begin{aligned} \mathrm{Ord}\, (AB^{-1}):=\mathrm{Ord}\,A-\mathrm{Ord}\, B,\quad \mathrm{Ord}\, ({\hat{B}}^{-1}{\hat{A}}):=\mathrm{Ord}\,{\hat{A}}-\mathrm{Ord}\,{\hat{B}},\qquad A,B,{\hat{A}}, \hat{B}\in {\mathcal {R}}. \end{aligned}$$

#### Definition 3

A formal adjoint operator $A^\dagger$ for any $A\in {\mathcal {Q}}$ can be defined recursively:

1. $$a^\dagger =a$$ for any $$a\in {\mathrm F}$$,
2. $${\mathcal S}^\dagger ={\mathcal S}^{-1}$$,
3. $$(A+ B)^\dagger =B^\dagger + A^\dagger $$ for any $$A,B\in {\mathcal {Q}}$$,
4. $$(A\cdot B)^\dagger =B^\dagger \cdot A^\dagger $$ for any $$A,B\in {\mathcal {Q}}$$,
5. $$(A^{-1})^\dagger =(A^\dagger )^{-1}$$ for any $$A\in {\mathcal {Q}}$$.

In particular, We say an operator $H\in {\mathcal {Q}}$ is skew-symmetric if $H^{\dagger }=-H$.

For example, we have

$$\begin{aligned} \left( ({\mathcal S}+a{\mathcal S}^{-1})\cdot (b-{\mathcal S})^{-1}\right) ^\dagger =(b-{\mathcal S}^{-1})^{-1}\cdot ({\mathcal S}^{-1}+{\mathcal S}(a){\mathcal S}),\qquad a,b\in {\mathrm F}. \end{aligned}$$

For any $A\in {\mathcal {Q}}$, if $\mathrm{ord}\, A=(p, q)$ then $\mathrm{ord}\,A^\dagger =(-q, -p)$. Obviously $\mathrm{Ord}\, A^\dagger =\mathrm{Ord}\, A$.

### Rational and weakly nonlocal difference operators

In the theory of integrable systems, the majority of $1+1$-dimensional integrable equations possesses weakly nonlocal [21] Nijenhuis recursion operators. For integrable differential–difference equations, weakly nonlocal operators are often rational operators with only a finite number of nonlocal terms of the form $a ({\mathcal S}-1)^{-1}b$, where $a, b \in {\mathrm F}$. In this section, we show how to write a weakly nonlocal operator as a rational operator and provide a way to test whether a rational operator is indeed weakly nonlocal. For the differential case, the answers are given by Lemma 4.5 in [17].

First we give a definition of the full kernel difference operators. We then prove that for such operators, their inverse are weakly nonlocal.

For a difference operator $A\in {\mathcal {R}}$ it is obvious that

16 $$\begin{aligned} \text{ dim }_{\mathrm k}\text{ Ker }\,A\le \mathrm{Ord}\, A. \end{aligned}$$

Indeed, if there is an element $a\in {\mathrm F}$ such that $a\in \text{ Ker }\,A$, then we can represent $A={\tilde{A}} ({\mathcal S}-1) \frac{1}{a},$ where $\mathrm{Ord}\,{\tilde{A}}=\mathrm{Ord}\,A-1$. Zero total order difference operator is invertible and thus it has a trivial kernel space. A difference operator of a nonzero order may also have a trivial kernel in ${\mathrm F}$ as well. For example $\text{ Ker }_{\mathrm k}({\mathcal S}-u)=0$ since equation ${\mathcal S}(v)=uv$ does not have a solution $v\in {\mathrm F}$.

#### Definition 4

We say that a difference operator has a full kernel in ${\mathrm F}$ (is a full kernel operator) if the dimension of its kernel over the field ${\mathrm k}$ equals to the total order of the operator.

In what follows, we show how to construct a full kernel operator given the generators of its kernel and prove an important property of such operators.

#### Proposition 6

Assume that $f^{(1)}, \ldots , f^{(n)}$ are linearly independent over ${\mathrm k}$ in ${\mathrm F}$. Then there exists a full kernel difference operator $P\in {\mathcal {R}}$ such that the $f^{(i)}, i=1, \ldots , n$ span $\ker P$.

#### Proof

We prove the statement by induction on *n*. If $n=1$, we define

$$\begin{aligned} P=({\mathcal S}-1)\frac{1}{f^{(1)}}. \end{aligned}$$

It is clear that ${\mathrm{Ord}}\,P=1$ and its kernel is spanned by $f^{(1)}$. Assume that *Q* is a full kernel operator with $\mathrm{Ord}\,Q=n-1$ and its kernel is spanned by $f^{(1)}, \ldots , f^{(n-1)}$. Since $f^{(i)}, i=1, \ldots , n$ are linearly independent, we have $Q(f^{(n)})\ne 0$ by construction of *Q*. We define

$$\begin{aligned} P=({\mathcal S}-1)\frac{1}{Q(f^{(n)})} Q. \end{aligned}$$

Clearly it is the required full kernel operator and its kernel is spanned by $f^{(1)}, \ldots , f^{(n)}$. $\quad \square$

#### Remark 2

A difference operator $Q\in {\mathcal {R}}$ with full kernel spanned by the ${\mathrm k}$–linearly independent elements $f^{(i)}\in {\mathrm F}, i=1, \ldots , n$, can be obtained using the determinant expression

$$\begin{aligned} Q(g)=\det \left( \begin{array}{c@{\quad }c@{\quad }c@{\quad }c} f^{(1)}&{}\cdots &{} f^{(n)}&{} g\\ {\mathcal S}( f^{(1)})&{}\cdots &{} {\mathcal S}( f^{(n)})&{}{\mathcal S}( g)\\ \vdots &{}\vdots &{}\vdots &{}\vdots \\ {\mathcal S}^n( f^{(1)})&{}\cdots &{} {\mathcal S}^n( f^{(n)})&{}{\mathcal S}^n( g) \end{array} \right) \ \text{ for } \text{ any } g\in {\mathrm F}. \end{aligned}$$

#### Proposition 7

The inverse operators of full kernel operators are weakly nonlocal.

#### Proof

We prove the statement by induced on the total order of such operator *B*. If *B* is a full kernel operator with $\mathrm{Ord}B=1$, it can be written as $B=a {\mathcal S}^i ({\mathcal S}-1) b$ for some $i\in \mathbb {Z}$. Thus

$$\begin{aligned} B^{-1}= \frac{1}{b} {\mathcal S}^{-i} ({\mathcal S}-1)^{-1} \frac{1}{a} \end{aligned}$$

is weakly nonlocal.

Let *B* be a full kernel operator with the total order of *n* and $a\in \ker B$. It follows from Proposition 6 that there is a full kernel operator *C* with total order of $n-1$ such that

$$\begin{aligned} B=C ({\mathcal S}-1)\frac{1}{a}. \end{aligned}$$

By the induction assumption, $C^{-1}$ is weakly nonlocal, that is, there exist two sets of linearly independent functions $b^{(i)}$ and $c^{(i)}$, $i=1,\ldots , n-1$ such that

$$\begin{aligned} C^{-1}=E+\sum _{i=1}^{n-1} b^{(i)} ({\mathcal S}-1)^{-1} c^{(i)}, \quad E\in {\mathcal {R}}. \end{aligned}$$

Multiplying *C* on its left, we get

$$\begin{aligned} \sum _{i=1}^{n-1} C(b^{(i)}) ({\mathcal S}-1)^{-1} c^{(i)}=0 \end{aligned}$$

implying $b^{(i)}\in \ker C.$ Note that for any $b^{(i)}\in \ker C$, $i=1,\ldots , n-1$, there exists $d^{(i)}$, which is in $\ker B$ such that $b^{(i)}=({\mathcal S}-1)\frac{d^{(i)}}{a}.$ Therefore, we have

$$\begin{aligned} B^{-1}= a ({\mathcal S}-1)^{-1} C^{-1}= a ({\mathcal S}-1)^{-1} \left( E+\sum _{i=1}^{n-1} \left( ({\mathcal S}-1)\frac{d^{(i)}}{a}\right) ({\mathcal S}-1)^{-1} c^{(i)}\right) , \end{aligned}$$

whose nonlocal terms are

$$\begin{aligned} a ({\mathcal S}-1)^{-1} E^{\dagger }(1) +\sum _{i=1}^{n-1} \left( d^{(i)} ({\mathcal S}-1)^{-1} c^{(i)}-a ({\mathcal S}-1)^{-1} \frac{c^{(i)} d_1^{(i)}}{a_1} \right) , \end{aligned}$$

where we used the identity

$$\begin{aligned} ({\mathcal S}-1)^{-1} (d_1-d) ({\mathcal S}-1)^{-1} =d ({\mathcal S}-1)^{-1} -({\mathcal S}-1)^{-1} d_1, \quad d\in {\mathrm F}. \end{aligned}$$

This leads to the conclusion that $B^{-1}$ is weakly nonlocal. $\quad \square$

We are now ready to prove the statement on the relation between the rational and weakly nonlocal difference operators.

#### Theorem 1

Let *R* be a rational operator with minimal right fractional decomposition $A B^{-1}$ and ${\mathrm{Ord}B}=n$. Then the following three statements are equivalent:

(i) The operator *B* has a full kernel in ${\mathrm F}$;
(ii) The operator *R* is weakly nonlocal, that is, $R=L+\sum _{i=1}^n p^{(i)} ({\mathcal S}-1)^{-1} q^{(i)}$, where $L\in {\mathcal {R}}$, and $\{p^{(i)}, i=1, \ldots , n\}$ and $\{q^{(i)},i=1,\ldots , n\}$ are two linearly independent sets over ${\mathrm k}$ in ${\mathrm F}$;
(iii) The operator $B^{\dagger }$ has a full kernel in ${\mathrm F}$.

#### Proof

The statement $\mathrm{(i)}\Rightarrow \mathrm{(ii)}$ directly follows from Proposition 7 since the multiplication of a difference operator and a weakly nonlocal operator is weakly nonlocal.

We now prove that $\mathrm{(ii)}\Rightarrow \mathrm{(iii)}$. Knowing

$$\begin{aligned} R=A B^{-1}=L+\sum _{i=1}^n p^{(i)}({\mathcal S}-1)^{-1} q^{(i)}, \end{aligned}$$

we multiply it on the right by *B* and obtain its nonlocal terms

$$\begin{aligned} \sum _{i=1}^n p^{(i)}\left( {\mathcal S}-1)^{-1} B^{\dagger }(q^{(i)}\right) =0, \end{aligned}$$

which implies that all $q^{(i)}$’s are in the kernel of $B^{\dagger }$ and thus $n\le \dim (\ker B^{\dagger })$.

Let *C* be a common multiple of the difference operators $\frac{1}{q^{(i)}} ({\mathcal S}-1)$, that is, a difference operator such that for all *i* there exists a difference operator $M^{(i)}$ satisfying $C=\frac{1}{q^{(i)}} ({\mathcal S}-1) M^{(i)}$. Thus we have

$$\begin{aligned} R=L+\sum _{i=1}^n p^{(i)}({\mathcal S}-1)^{-1} q^{(i)}=(LC+\sum _{i=1}^n p^{(i)}M^{(i)})C^{-1}. \end{aligned}$$

Since $AB^{-1}$ is a minimal right fractional decomposition for *R*, there exists a difference operator *D* such that

$$\begin{aligned} LC+\sum _{i=1}^n p^{(i)}M^{(i)}=AD \quad \text{ and } \quad C=BD. \end{aligned}$$

This leads to $\mathrm{Ord}C=n\ge \mathrm{Ord}B$. Note that $\mathrm{Ord}B=\mathrm{Ord}B^{\dagger }$ and $\mathrm{Ord}B^{\dagger }\ge \dim (\ker B^{\dagger })$. Therefore, we have

$$\begin{aligned} \mathrm{Ord}B^{\dagger }=\dim (\ker B^{\dagger })=n \end{aligned}$$

implying that $B^{\dagger }$ has a full kernel spanned by all $q^{(i)}$’s.

Finally we prove that $\mathrm{(iii)}\Rightarrow \mathrm{(i)}$. It follows from Proposition 7 that the inverse of $B^{\dagger }$ is weakly nonlocal. Using the proof of $\mathrm{(ii)}\Rightarrow \mathrm{(iii)}$, we obtain that statement of $\mathrm{(i)}$. $\quad \square$

From the proof of Theorem 1, we are able to specify the nonlocal terms for weakly nonlocal operator.

#### Corollary 2

Under the condition of Theorem 1, for $R=L+\sum _{i=1}^n p^{(i)} ({\mathcal S}-1)^{-1} q^{(i)}$, the linearly independent functions $p^{(i)}$’s span $A(\ker B)$ and the linearly independent functions $q^{(i)}$’s span $\ker B^\dagger$, $i=1, \ldots , n$.

Following from this theorem, we are immediately able to get the statement for the inverse of rational operator:

#### Corollary 3

Let $R=AB^{-1}$ with $A, B\in {\mathcal {R}}$. Then $R^{-1}$ is weakly nonlocal if and only if *A* has a full kernel in ${\mathrm F}$.

Corollary 2 combined with Proposition 6 provides us with a method to write a weakly nonlocal operator in the form of a rational operator $R=AB^{-1}$: We first construct a full kernel operator $B^\dagger$ using $q^{(i)}$’s. Then we have $A=RB$. We use such construction for the examples in Sect. 6, where we will also apply Corollary 3 to the recursion operators of integrable differential–difference equations to see whether their inverse operators are weakly nonlocal or not. If so, we are going to compute the seeds for symmetry and co–symmetry hierarchies (its nonlocal terms), that is, the $p^{(i)}$’s and $q^{(i)}$’s for $R^{-1}$ in the above theorem.

### Matrix difference and rational pseudo–difference operators

We recall here some facts from linear algebra over non-commutative rings and skew fields, which is a specialisation of the general theory [22, 23] to the case of difference algebra (the ring ${\mathcal {R}}$ and skew field ${\mathcal {Q}}$). We denote by ${\mathcal M}_n({\mathcal {R}})$ and ${\mathcal M}_n({\mathcal {Q}})$ the rings of $n\times n$ matrices over the ring ${\mathcal {R}}$ and skew field ${\mathcal {Q}}$ respectively. Since ${\mathcal {R}}$ is a principal ideal ring, then the ring ${\mathcal M}_n({\mathcal {R}})$ is also a principal ideal ring (see proof in [24], as well as the short and useful review of non-commutative principal ideal rings [25]).

Let ${\mathcal {A}}_i$ denote the *i*–th row of the matrix ${\mathcal {A}}$ and ${\mathcal {A}}_{i,j}$ denote the (*i*, *j*) entry of ${\mathcal {A}}$. For $1\le i\ne j\le n$ and arbitrary $B\in {\mathcal {R}}$ (or $B\in {\mathcal {Q}}$) the ${\mathcal {R}}$–elementary (resp. ${\mathcal {Q}}$–elementary) row operation $\tau _{i,j}(B)$ changes the row ${\mathcal {A}}_i\mapsto {\mathcal {A}}_i +B\cdot {\mathcal {A}}_j$ and leaves the other rows unchanged. The transformation $\tau _{i,j}(B)$ is invertible ($\tau _{i,j}(B)\tau _{i,j}(-B)=\mathrm{Id}$) and can be represented by a multiplication from the left by the matrix $\tau _{i,j}(B)=I+B E_{i,j}$, where *I* is the unit matrix and $E_{i,j}$ is the matrix with the (*i*, *j*) entry equal to 1 and zero elsewhere. Note that the transformation $\sigma _{i,j}=\tau _{i,j}(1) \tau _{j,i}(-1)\tau _{i,j}(1)$ replaces ${\mathcal {A}}_i$ by ${\mathcal {A}}_j$ and ${\mathcal {A}}_j$ by $-{\mathcal {A}}_i$, leaving other rows unchanged.

${\mathcal {R}}$–elementary row operations generate a group ${\mathcal E}_n({\mathcal {R}})$, which is a subgroup of the group $GL_n({\mathcal {R}})$ of invertible matrix difference operators. Similarly, ${\mathcal {Q}}$–elementary row operations generate a group ${\mathcal E}_n({\mathcal {Q}})$, a subgroup of the group $GL_n({\mathcal {Q}})$ of invertible matrix pseudo–difference operators.

#### Lemma 2

Let ${\mathcal {A}}\in {\mathcal M}_n({\mathcal {R}})$. Then there exist two invertible matrices $\mathcal {U}$ and $\mathcal {V}$ such that $\mathcal {U}{\mathcal {A}}\mathcal {V}$ is diagonal.

#### Proof

Let $\mathcal {N}$ be an element of the set $E=\{ \mathcal {U}{\mathcal {A}}\mathcal {V} | \mathcal {U}, \mathcal {V} \in {\mathcal E}_n({\mathcal {R}})\}$ such that for all ${\mathcal M}\in E$, either ${\mathcal M}_{11}=0$ or $\mathrm{Ord}\, \mathcal {N}_{11} \le \mathrm{Ord}\, {\mathcal M}_{11}$. We claim that all entries in the first column of $\mathcal {N}$ are divisible on the right by $\mathcal {N}_{11}$. Otherwise, using elementary row operations which amounts to multiply $\mathcal {N}$ on the left by an invertible matrix, one can find ${\mathcal M}\in E$ such that ${\mathcal M}_{11} \ne 0$ and $\mathrm{Ord}\, {\mathcal M}_{11}< \mathrm{Ord}\, \mathcal {N}_{11}$, which contradicts the definition of $\mathcal {N}$. Similarly, $\mathcal {N}_{11}$ must divide all the entries of the first row of $\mathcal {N}$ on the left. Therefore, there exist invertible matrix difference operators $\mathcal {U}$ and $\mathcal {V}$ such that $\mathcal {U}\mathcal {N}\mathcal {V}$ has only zero entries in its first row and first column, apart from the first coefficient which is $\mathcal {N}_{11}$. We conclude by induction on *n*. $\quad \square$

#### Proposition 8

Let $${\mathcal {A}}\in {\mathcal M}_n({\mathcal {R}})$$. Then it can be brought to a upper triangular form $$ {\mathcal {A}}^\vartriangle $$ with $${\mathcal {A}}_{i,j}^\vartriangle =0$$ for $$ i>j$$ by $${\mathcal {R}}$$–elementary row operations and

$$\begin{aligned} {\mathcal {A}}^\vartriangle ={\mathcal {G}}{\mathcal {A}},\quad {\mathcal {G}}\in {\mathcal E}_n({\mathcal {R}}). \end{aligned}$$

#### Proof

We prove the claim by induction on *n*. If $n=1$, the matrix is already in the form required. Now we assume that any matrix from ${\mathcal M}_{n-1}({\mathcal {R}})$ can be brought to a upper triangular form by ${\mathcal {R}}$–elementary row transformation. Therefore the first $n-1$ rows of matrix ${\mathcal {A}}$ can be brought to the upper triangular form.

(i) If ${\mathcal {A}}_{n,1}=0$, then by deleting the first row and the first column of ${\mathcal {A}}$ we reduce the problem to the case ${\mathcal M}_{n-1}({\mathcal {R}})$ and we are done due to the induction hypothesis.
(ii) If ${\mathcal {A}}_{1,1}=0$, we use the transformation $\sigma _{1,n}$ to reduce the problem to the case (i).
(iii) The remaining case are ${\mathcal {A}}_{1,1}\ne 0,\ {\mathcal {A}}_{n,1}\ne 0$. Suppose $\mathrm{Ord}\, {\mathcal {A}}_{n,1}\le \mathrm{Ord}\, {\mathcal {A}}_{1,1}$ (otherwise, we can swap the rows by the transformation $\sigma _{1,n}$). Then there exist $B,R\in {\mathcal {R}}$ such that ${\mathcal {A}}_{1,1}=B\cdot {\mathcal {A}}_{n,1}+R$ and either $R=0$ or $\mathrm{Ord}\, R<\mathrm{Ord}\, {\mathcal {A}}_{n,1}$ and we apply the transformation $\tau _{1,n}(-B)$ replacing ${\mathcal {A}}_1$ by ${\hat{{\mathcal {A}}}}_1={\mathcal {A}}_1-B{\mathcal {A}}_n$. If $R=0$, then the updated row ${\hat{{\mathcal {A}}}}_{1}$ has zero entry ${\hat{{\mathcal {A}}}}_{1,1}=0$ and we are done (ii), or $\mathrm{Ord}\, {\hat{{\mathcal {A}}}}_{1,1}< \mathrm{Ord}\, {\mathcal {A}}_{n,1}$ and we use $\sigma _{1,n}$ to swap the rows. Iterating this procedure we can make the entry (*n*, 1) vanish, reducing the problem to the first case (i). $\quad \square$

The ring ${\mathcal M}_{n}({\mathcal {R}}), n\ge 2$ has zero divisors. We will denote by ${\mathcal M}_{n}^\times ({\mathcal {R}})$ the multiplicative monoid of regular elements, i.e. the elements which are not zero divisors. A difference matrix operator ${\mathcal {A}}$ is *regular* if and only if its upper triangular form ${\mathcal {A}}^\vartriangle$ is regular, i.e. if and only if

17 $$\begin{aligned} \delta \epsilon \tau ({\mathcal {A}}^\vartriangle ):={\mathcal {A}}_{1,1}^\vartriangle \cdot {\mathcal {A}}_{2,2}^\vartriangle \cdots {\mathcal {A}}_{n,n}^\vartriangle \ne 0. \end{aligned}$$

#### Definition 5

The total order of a matrix difference operator ${\mathcal {A}}\in {\mathcal M}_{n}({\mathcal {R}})$ is defined as the sum of total orders of the diagonal entries of a corresponding upper triangular operator ${\mathcal {A}}^\vartriangle$, i.e.

$$\begin{aligned} \mathrm{Ord}\, {\mathcal {A}}=\sum _{i=1}^n \mathrm{Ord}\, {\mathcal {A}}^\vartriangle _{i,i}=\mathrm{Ord}\, \delta \epsilon \tau ({\mathcal {A}}) . \end{aligned}$$

#### Proposition 9

A difference matrix operator ${\mathcal {A}}$ is invertible in ${\mathcal M}_{n}({\mathcal {R}})$ (i.e. ${\mathcal {A}}^{-1}\in {\mathcal M}_{n}({\mathcal {R}})$ and thus ${\mathcal {A}}\in GL_n({\mathcal {R}})$), if and only if $\mathrm{Ord}\, {\mathcal {A}}=0$.

#### Proof

If $\mathrm{Ord}\, {\mathcal {A}}=0$, then all entries on the diagonal part ${\mathcal {A}}^\vartriangle _d:=\mathrm{diag}(({\mathcal {A}}_{1,1}^\vartriangle ) ,\ldots , ({\mathcal {A}}_{n,n}^\vartriangle ))$ of ${\mathcal {A}}^\vartriangle$ have total order zero and thus invertible. Multiplying ${\mathcal {A}}^\vartriangle$ on the left by matrix $({\mathcal {A}}^\vartriangle _d)^{-1}$ we obtain an upper triangular matrix $\tilde{{\mathcal {A}}}^\vartriangle = ({\mathcal {A}}^\vartriangle _d)^{-1}{\mathcal {G}}{\mathcal {A}}$ with the unit matrix on the diagonal. By induction on *n* it is easy to show that there is a composition of ${\mathcal {R}}$–elementary row transformations ${\tilde{{\mathcal {G}}}}$ such that ${\tilde{{\mathcal {G}}}} \tilde{{\mathcal {A}}}^\vartriangle =I$. If $n=1$ there is nothing to do. We assume the existence of the inverse matrix in ${\mathcal M}_{n-1}({\mathcal {R}})$. The entries $\tilde{{\mathcal {A}}}^\vartriangle _{k,n},\ k=1,\ldots ,n-1$ of the last column can be set to zero by the transformation $\prod _{k=1}^{n-1}\tau _{k,n}(-\tilde{{\mathcal {A}}}^\vartriangle _{k,n}) \cdot \tilde{{\mathcal {A}}}^\vartriangle$ which reduces the problem to the case in ${\mathcal M}_{n-1}({\mathcal {R}})$. The necessity is obvious from the consideration of a diagonal matrix ${\mathcal {A}}$. $\quad \square$

#### Example 1

Let us consider the following matrix difference operator

18 $$\begin{aligned} {\mathcal {A}}=\left( \begin{array}{c@{\quad }c} 1&{}{\mathcal S}^2\\ a_{-1}{\mathcal S}^{-1}&{}a_1{\mathcal S}\end{array}\right) , \end{aligned}$$

where $a\in {\mathrm F},\ a_k={\mathcal S}^k(a)$ and $a_i\ne a_{j}$ if $i\ne j$. The transformation ${\mathcal {A}}\mapsto {\mathcal {A}}^\vartriangle =\tau _{2,1}(-a_{-1}{\mathcal S}^{-1}){\mathcal {A}}$ brings ${\mathcal {A}}$ to an upper triangular form and $\delta \epsilon \tau ({\mathcal {A}}^\vartriangle )=(a_1-a_{-1}){\mathcal S}$. Thus $\mathrm{Ord}\, {\mathcal {A}}=0$ and the inverse matrix difference operator of ${\mathcal {A}}$ exists. Indeed,

$$\begin{aligned} {\mathcal {A}}^{-1}=\left( \begin{array}{c@{\quad }c@{\quad }c} \dfrac{a_2}{a_2-a}&{}\ &{}-\dfrac{1}{a_2-a}{\mathcal S}\\ {} &{} &{}\\ -\dfrac{a_{-2}}{a-a_{-2}}{\mathcal S}^{-2}&{}\ &{}\dfrac{1}{a-a_{-2}}{\mathcal S}^{-1} \end{array}\right) . \end{aligned}$$

If we use a different sequence of elementary row transformations

$$\begin{aligned} {\mathcal {A}}\mapsto \tilde{\mathcal {A}}^\vartriangle =\tau _{1,2}\left( -\frac{1}{a_{2}}{\mathcal S}\right) \tau _{2,1}\left( -\frac{a_1 a_{-1}}{a_1-a_{-1}}{\mathcal S}^{ -1 }\right) {\mathcal {A}}, \end{aligned}$$

which also brings the difference matrix operator ${\mathcal {A}}$ to an upper triangular form, then $\delta \epsilon \tau ({\tilde{{\mathcal {A}}}}^\vartriangle )=\frac{a_1(a_2-a)}{a_2}{\mathcal S}$, but the total order of ${\mathcal {A}}$ does not depend on the choice of the sequence $\mathrm{Ord}\, A=\mathrm{Ord}\, \delta \epsilon \tau ({\tilde{{\mathcal {A}}}}^\vartriangle )=0$ (see below).

The correctness of Definition 5, i.e. the independence of $\mathrm{Ord}\,{\mathcal {A}}$ from the choice of row transformations, can be justified by the theory of Dieudonné determinants ($\Delta _n$) (in the case of skew polynomial rings it has been discussed in [26]). The above definition of total order for matrix difference operators is a restriction of the map $\mathrm{Ord}\,\Delta _n\,:{\mathcal M}_n({\mathcal {Q}})\mapsto {\mathbb {Z}}\cup \{\infty \}$ to $\mathrm{Ord}\,\Delta _n\,:{\mathcal M}_n({\mathcal {R}})\mapsto {\mathbb {Z}}_{\ge 0}\cup \{\infty \}$. This observation results in a simpler way to compute the total order of matrix difference operators by treating them as elements of ${\mathcal M}_n({\mathcal {Q}})$.

The Dieudonné determinant $\Delta _n$ is defined for matrices with entries in an arbitrary skew field ${\mathbb {K}}$ (see [22, 23, 27]). In our case the skew field is ${\mathbb {K}}={\mathcal {Q}}$ and we are dealing with matrix rational operators ${\mathcal M}_n({\mathcal {Q}})$, but what is presented below is equally applicable to rational operators or any skew field of fraction of a left principal ideal domain. The Dieudonné determinant is a map from ${\mathcal M}_{n}({\mathcal {Q}})$ to ${\bar{{\mathcal {Q}}}}={\mathcal {Q}}^\times \diagup {\mathcal {Q}}^{ (1)}$ or zero, where ${\mathcal {Q}}^\times$ is the multiplicative group of nonzero elements of ${\mathcal {Q}}$, and ${\mathcal {Q}}^{(1)}$ denotes the commutator subgroup ${\mathcal {Q}}^{(1)}=[{\mathcal {Q}}^\times ,{\mathcal {Q}}^\times ]\subset {\mathcal {Q}}^\times$, which is normal. The group ${\mathcal {Q}}^{ (1)}$ is generated by elements of the form $ABA^{-1}B^{-1},\ A,B\in {\mathcal {Q}}^\times$. The quotient group ${\bar{{\mathcal {Q}}}}$ is commutative and its elements are cosets $A{\mathcal {Q}}^{ (1)},\ A\in {\mathcal {Q}}^{\times }$. There is a natural projection $\pi \, : \, {\mathcal {Q}}^\times \mapsto {\bar{{\mathcal {Q}}}}$ given by $\pi (A)={\bar{A}}:=A{\mathcal {Q}}^{ (1)}$ for any $A\in {\mathcal {Q}}^\times$.

Dieudonné has shown that ${\mathcal E}_n({\mathcal {Q}})$ is a normal subgroup of $GL_n({\mathcal {Q}})$ and that there is a group isomorphism $\Delta _n: GL_n({\mathcal {Q}})\diagup {\mathcal E}_n({\mathcal {Q}})\mapsto {\bar{{\mathcal {Q}}}}$ given by a map $\Delta _n$ (Theorem 1. in [27]), which is now called the Dieudonné determinant. The function $\Delta _n : {\mathcal M}_n({\mathcal {Q}})\mapsto {\bar{{\mathcal {Q}}}}$ is:

1. multiplicative: $$\Delta _n ({\mathcal {A}}{\mathcal {B}})=\Delta _n({\mathcal {A}})\Delta _n({\mathcal {B}})$$;
2. if $${\mathcal {A}}\in {\mathcal E}_n({\mathcal {Q}})$$, then $$\Delta _n({\mathcal {A}})={\bar{1}}$$;
3. if ${\mathcal {A}}'$ is obtained from ${\mathcal {A}}$ by multiplying one row of ${\mathcal {A}}$ on the left by $B\in {\mathcal {Q}}$, then
  $$\begin{aligned} \Delta _n {\mathcal {A}}'={\bar{B}}\,\cdot \,\Delta _n{\mathcal {A}}; \end{aligned}$$
4. if a matrix ${\mathcal {A}}$ is degenerate (i.e. one row is a left ${\mathcal {Q}}$–linear combination of other rows), then $\Delta _n({\mathcal {A}})=0$.

In order to find $\Delta _n {\mathcal {A}}$ for ${\mathcal {A}}\in {\mathcal M}_n({\mathcal {Q}})$ one can use the algorithm given by Dieudonné [27] (see also §1, Ch. IV [22]), or use the Bruhat normal form approach (§20, Part III, [23]). A simple way to find the Dieudonné determinant of a matrix ${\mathcal {A}}\in {\mathcal M}_n({\mathcal {Q}})$ is to use a composition of ${\mathcal {Q}}$–elementary row transformations in order to bring the matrix ${\mathcal {A}}$ to a upper triangular form ${\mathcal {A}}^\vartriangle ={\mathcal {G}}{\mathcal {A}},\ {\mathcal {G}}\in {\mathcal E}_n({\mathcal {Q}})$, then multiply the diagonal entries of ${\mathcal {A}}^\vartriangle$ (in an arbitrary order) and apply the projection $\pi$ to the result

$$\begin{aligned} \Delta _n({\mathcal {A}})=\pi \left( \prod _{k=1}^n {\mathcal {A}}^\vartriangle _{k,k}\right) . \end{aligned}$$

It follows from [27] that $\Delta _n({\mathcal {A}})$ does not depend on the choice of elementary row transformations, neither on the order in the product of diagonal elements of ${\mathcal {A}}^\vartriangle$.

It follows from Definition 1 and (15) that $\mathrm{Ord}P=0$ for any $P\in {\mathcal {Q}}^{ (1)}$, thus function $\mathrm{Ord}$ has a constant value on a coset and the map

$$\begin{aligned} \mathrm{Ord}\, :\, {\bar{{\mathcal {Q}}}} \ \mapsto \ {\mathbb {Z}}\end{aligned}$$

is defined correctly.

#### Definition 6

The total order of a matrix rational operator ${\mathcal {A}}\in {\mathcal M}_n({\mathcal {Q}})$ is

$$\begin{aligned} \mathrm{Ord}\,{\mathcal {A}}:=\mathrm{Ord}\,\Delta _n({\mathcal {A}}). \end{aligned}$$

In the case of difference operators ${\mathcal {A}}\in {\mathcal M}_n({\mathcal {R}})$ we have defined a function $\delta \epsilon \tau ({\mathcal {A}}^\vartriangle )\in {\mathcal {R}}$ (17). Although the value of this function depends on the choice of ${\mathcal {R}}$–elementary row transformations, its natural projection to ${\bar{{\mathcal {Q}}}}$ does not, since it coincides with the Dieudonné determinant

$$\begin{aligned} \pi (\delta \epsilon \tau ({\mathcal {A}}^\vartriangle ))=\Delta _n({\mathcal {A}}). \end{aligned}$$

This restriction of the total order definition to the ring of matrix difference operators together with Proposition 9 results in the exact sequence of monoid homomorphisms (similar to Theorem 1.1 in [26]):

$$\begin{aligned} 1\ \longmapsto \ GL_n({\mathcal {R}})\ \longmapsto \ {\mathcal M}_n({\mathcal {R}})\ \longmapsto \ {\mathbb {Z}}_{\ge 0}\cup \{\infty \}\ \longmapsto \ 0\ . \end{aligned}$$

Definition 5 is a way to define the total order of a matrix difference operator, bypassing the skew field of rational operators, its quotient group ${\bar{{\mathcal {Q}}}}$ and the theory of Dieudonné determinants.

Note that the Dieudonné determinant and the total order of a matrix (rational) difference operator and the transposed matrix operator may not coincide. In the above Example (18):

$$\begin{aligned} \Delta _2({\mathcal {A}})=\pi ((a_1-a_{-1}){\mathcal S}),\qquad \Delta _2({\mathcal {A}}^{tr})=0. \end{aligned}$$

A formally conjugated matrix (rational) difference operator has a usual definition, i.e. the corresponding matrix is transposed and each entry is formally conjugated: $({\mathcal {A}}^\dagger )_{i,j}=({\mathcal {A}}_{j,i})^\dagger$. For formally conjugated operators we have $\Delta _n({\mathcal {A}}^\dagger )=(\Delta _n {\mathcal {A}})^\dagger$ and therefore $\mathrm{Ord}{\mathcal {A}}^\dagger =\mathrm{Ord}{\mathcal {A}}$.

There are many ways to represent a matrix rational operator as a ratio of matrix difference operators. For example any ${\mathcal {L}}\in {\mathcal M}_n({\mathcal {Q}})$ can be represented as

$$\begin{aligned} {\mathcal {L}}={\hat{{\mathcal {A}}}}\cdot {\mathcal {D}}^{-1}={\tilde{{\mathcal {A}}}}\cdot M^{-1},\quad {\mathcal {D}}=\text{ diag } (M_1,\ldots ,M_n),\ M_k,M\in {\mathcal {R}}{\setminus }\{0\}. \end{aligned}$$

Indeed, the entries ${\mathcal {L}}_{i,j}\in {\mathcal {Q}}$ and thus ${\mathcal {L}}_{i,j}=A_{i,j}B_{i,j}^{-1},\ A_{i,j},B_{i,j}\in {\mathcal {R}}$. Since the ring ${\mathcal {R}}$ satisfies the Ore property (Proposition 2) there exists a least right common multiple $M_i$ of the elements $B_{1,i},\ldots B_{n,i}$ and therefore there exist $P_{1,i},\ldots P_{n,i}\in {\mathcal {R}}$ such that $M_i=B_{1,i}P_{1,i}=\cdots =B_{n,i}P_{n,i}$. Taking ${\hat{{\mathcal {A}}}}_{i,j}={\mathcal {L}}_{i,j}P_{i,j}$ we obtain the first representation. Let *M* be the least right common multiple of $M_1,\ldots ,M_n$. There exist $Q_1,\dots Q_n\in {\mathcal {R}}$ such that $M=M_1 Q_1=\cdots =M_nQ_n$, therefore $\tilde{A}=\hat{A}\cdot \text{ diag }(Q_1,\ldots ,Q_n)$.

Since the ring of difference operators ${\mathcal {R}}$ is a principal ideal domain, the ring of matrices ${\mathcal M}_n({\mathcal {R}})$ satisfies the left and right Ore property (see proof in [24]) and thus

$$\begin{aligned} \begin{aligned} \mathcal {M}_{n}({\mathcal {Q}}) =&\{{\mathcal {A}}{\mathcal {B}}^{-1}|({\mathcal {A}},{\mathcal {B}}) \in \mathcal {M}_{n}({\mathcal {R}}) \times \mathcal {M}_{n}^{\times }({\mathcal {R}})\} \\ =&\{{\mathcal {B}}^{-1}{\mathcal {A}}| ({\mathcal {A}},{\mathcal {B}}) \in \mathcal {M}_{n}({\mathcal {R}}) \times \mathcal {M}_{n}^{\times }({\mathcal {R}})\}. \end{aligned} \end{aligned}$$

A representation of matrix rational operators as right (left) fractions is not unique. However, once we clear the common right (resp. left) divisors, we get a minimal fraction, in the following sense:

#### Theorem 2

For any ${\mathcal {L}}\in \mathcal {M}_{n}({\mathcal {Q}})$ there is a minimal right (resp. left) decomposition ${\mathcal {L}}={\mathcal {A}}{\mathcal {B}}^{-1}$ (resp. ${\mathcal {L}}={\hat{{\mathcal {B}}}}^{-1}{\hat{{\mathcal {A}}}}$) with ${\mathcal {A}},{\mathcal {B}}$ right (resp. ${\hat{{\mathcal {A}}}},{\hat{{\mathcal {B}}}}$ left) coprime. Any other right decomposition ${\mathcal {L}}={\mathcal {A}}_1{\mathcal {B}}_1^{-1}$ (resp. left decomposition ${\mathcal {L}}={\hat{{\mathcal {A}}}}_1^{-1}{\hat{{\mathcal {B}}}}_1$) is of the from ${\mathcal {A}}_1={\mathcal {A}}\cdot {\mathcal {C}},\ {\mathcal {B}}_1={\mathcal {B}}\cdot {\mathcal {C}}$ (resp. ${\hat{{\mathcal {A}}}}_1={\mathcal {C}}\cdot {\hat{{\mathcal {A}}}},\ {\mathcal {B}}_1={\mathcal {C}}\cdot {\hat{{\mathcal {B}}}}$), where ${\mathcal {C}}\in {\mathcal M}_n^\times ({\mathcal {R}})$. Moreover $\mathrm{Ord}\, {\mathcal {B}}=\mathrm{Ord}\,{\hat{{\mathcal {B}}}}$ and is minimal possible among all decompositions.

#### Proof

We will first prove by induction on *n* that if *A* and *B* are matrix difference operators of size $n \times n$ with *B* regular, if *M* is a generator of the right ideal $A{\mathcal M}_n({\mathcal {R}}) \cap B {\mathcal M}_n({\mathcal {R}})$ and *N* a greatest left common divisor of *A* and *B*, then $\mathrm{Ord}A+ \mathrm{Ord}B=\mathrm{Ord}M+\mathrm{Ord}N$.

It is true for $n=1$ by Proposition 2. Let us now consider *A* and *B* of size $n+1$. Using invertible matrices we can assume that *A* and *B* are both upper triangular. Indeed, one can factorize them as $A=T_AU_A$ and $B=T_BU_B$ with $T_A,T_B$ upper triangular and $U_A$, $U_B$ invertible. Hence if there exist *C* and *D* such that $T_AD=T_BC$ with $\mathrm{Ord}D \le \mathrm{Ord}T_B=\mathrm{Ord}B$, then we can write $A(U_A^{-1}D)=B(U_B^{-1}C)$. Let us consider *A* and *B* in block matrix form:

$$\begin{aligned} A= \left( {\begin{array}{*{5}c} E &{} X \\ 0 &{} P \end{array}} \right) , \quad B= \left( {\begin{array}{*{5}c} F &{} Y \\ 0 &{} Q \end{array}} \right) , \end{aligned}$$

where *E* and *F* are of size $n \times n$, *P* and *Q* are difference operators and *X* and *Y* have size $n \times 1$. First, let $EG=FH$ be a generator of the right ideal $E{\mathcal M}_n({\mathcal {R}}) \cap F {\mathcal M}_n({\mathcal {R}})$ in ${\mathcal M}_n({\mathcal {R}})$, $P {\hat{Q}}=Q \hat{P}$ be a generator of the right ideal $P {\mathcal {R}}\cap Q {\mathcal {R}}$ in ${\mathcal {R}}$ and *K* be a generator of the right ideal $E{\mathcal M}_n({\mathcal {R}})+ F {\mathcal M}_n({\mathcal {R}})$ in ${\mathcal M}_n({\mathcal {R}})$ (which is also called the greatest left common divisor of *E* and *F*). We have by the induction hypothesis $\mathrm{Ord}K=\mathrm{Ord}F-\mathrm{Ord}G$. One can find a difference operator *R* with $\mathrm{Ord}R \le \mathrm{Ord}K$ and a vector difference operator *Z* such that $KZ=(Y {\hat{P}}-X {\hat{Q}})R$. Indeed, by Lemma 2 one can assume that *K* is a diagonal matrix $diag(K_0,\ldots ,K_{n})$. Let us call by $L_0,\ldots ,L_n$ the entries of the vector $Y {\hat{P}}-X {\hat{Q}}$. Then we can find for all $i=0,\ldots n$ difference operators $M_i$ and $N_i$ such that $\mathrm{Ord}N_i \le \mathrm{Ord}K_i$ and $K_iM_i=L_iN_i$. Let *R* be a generator of the right ideal $N_0 {\mathcal {R}}\cap \cdots \cap N_n {\mathcal {R}}$. Then $\mathrm{Ord}R \le \sum _{i=0}^n{\mathrm{Ord}N_i} \le \sum _{i=0}^n{\mathrm{Ord}K_i}=\mathrm{Ord}K$ and there exists a vector *Z* such that $KZ=LR$. Finally, by definition of *K* there exist two matrix difference operator *V* and *W* such that $EV-FW=K$. Let

$$\begin{aligned} C= \left( {\begin{array}{*{5}c} H &{} WZ \\ 0 &{} {\hat{P}} R \end{array}} \right) , \quad D= \left( {\begin{array}{*{5}c} G &{} VZ \\ 0 &{} {\hat{Q}} R \end{array}} \right) . \end{aligned}$$

Then $$\mathrm{Ord}D \le \mathrm{Ord}B$$ and $$AD=BC$$.

The proof of the remaining parts of the statement are identical to the scalar case, see the proofs of Propositions 2 and 5. $\quad \square$

The inequality (16) is also true for a regular matrix difference operator ${\mathcal {A}}\in {\mathcal M}_n^\times ({\mathcal {R}})$ and we say that ${\mathcal {A}}$ is a full kernel operator if $\text{ Dim }_{\mathrm k}\text{ Ker }\, {\mathcal {A}}=\mathrm{Ord}\,{\mathcal {A}}$. Theorem 1, Corollary 2 and Corollary 3 from the previous section are also true for matrix rational operators.

## PreHamiltonian Pairs and Nijenhuis Operators

Zhiber and Sokolov, in their study of Liouville integrable hyperbolic equations [28], have discovered a family of special differential operators with the property that they define a new Lie bracket and are homomorphisms from the Lie algebra with the newly induced bracket to the original Lie algebra. These operators can be viewed as a generalization of Hamiltonian operators, although they are not necessarily skew–symmetric. Inspired by the work of Zhiber and Sokolov, infinite sequences of such scalar differential operators of arbitrary order were constructed in [29] using symbolic representation [30, 31]. Kiselev and van de Leur gave some examples of such matrix differential operators [32] and investigated the geometric meaning of such operators. They named them preHamiltonian operators in [33] and defined the compatibility of two such operators. Recently, Carpentier renamed them as integrable pairs and investigated the interrelations between such pairs and Nijenhuis operators [17]. In principle, many results for differential operators also work for difference operators since ${\mathcal {R}}$ is a principal ideal domain. In this section, we develop further the theory of preHamiltonian operators and extend it to the difference case. Similarly to the previous section, we illustrate our results for the scalar case.

### Definition 7

A difference operator *A* is called preHamiltonian if ${\text {Im}}A$ is a Lie subalgebra of $({\mathrm F},[ \bullet , \bullet ])$, i.e. if

19 $$\begin{aligned} {[}{\text {Im}}A,\ {\text {Im}}A]\subseteq {\text {Im}}A . \end{aligned}$$

By a direct computation, it is easy to see ([29]) that an operator *A* is preHamiltonian if and only if there exists a 2-form on ${\mathrm F}$ denoted by $\omega _{A}$ such that

20 $$\begin{aligned} A_*[A a](b)-A_*[A b](a)=A \omega _{A}(a,b)\quad \text{ for } \text{ all } \quad a,b \in {\mathrm F}. \end{aligned}$$

For a given $a \in {\mathrm F}$, both $\omega _{A}(a, \bullet )$ and $\omega _{A}(\bullet , a)$ are in ${\mathcal {R}}$, i.e. difference operators on ${\mathrm F}$.

For a Hamiltonian operator *H*, the Jacobi identity is equivalent to (cf. [9])

21 $$\begin{aligned} {[}H a, H b]=H\left( b_*[H a]+(Ha)_*^{\dagger }(b)-a_*[H b]+a_*^{\dagger }(Hb)\right) , \end{aligned}$$

for all $a, b \in {\mathrm F}$, where $\dagger$ is the adjoint of the operator. Clearly, Hamiltonian operators are preHamiltonian with $\omega _H(a, b)=(Ha)_*^{\dagger }(b)+a_*^{\dagger }(Hb).$ We are going to explore the relation between preHamiltonian pairs and Hamiltonian pairs in the forthcoming paper [34]. Here we look at their relations with Nijenhuis operators.

Similarly to Hamiltonian operators, in general, the linear combination of two preHamiltonian operators is no longer preHamiltonian. This naturally leads to the following definition:

### Definition 8

We say that two difference operators *A* and *B* form a preHamiltonian pair if $A+\lambda B$ is preHamiltonian for all constant $\lambda \in {\mathrm k}$.

A preHamiltonian pair *A* and *B* implies the existence of 2-forms $\omega _A$, $\omega _B$ and $\omega _{A+\lambda B}=\omega _A +\lambda \omega _B$. They satisfy

22 $$\begin{aligned}&A_*[B a](b) +B_*[A a](b)-A_*[B b](a)-B_*[A b](a)\nonumber \\&\quad =A \omega _B(a,b)+B \omega _A(a,b) \ \text{ for } \text{ all } \ a,b \in {\mathrm F}. \end{aligned}$$

Gel’fand and Dorfman [8] and Fuchssteiner and Fokas [10, 11] discovered the relations between Hamiltonian pairs and Nijenhuis operators. These pairs naturally generate Nijenhuis operators. In what follows, we show that preHamiltonian pairs also give rise to Nijenhuis operators. This also explains why we chose the terminology ‘preHamiltonian’ instead of ‘integrable’ for such operators. These operators naturally appear in the description of the invariant evolutions of curvature flows [35].

### Definition 9

A difference operator *R* is Nijenhuis if

23 $$\begin{aligned} {[}R a, \ R b]-R[R a, b]-R[a, R b]+R^2[a,b]=0 \quad \text{ for } \text{ all } \quad a,b \in {\mathrm F}. \end{aligned}$$

Clearly, a Nijenhuis operator is also preHamiltonian with

$$\begin{aligned} \omega _R (a,b)=(Rb)_*[a]-(R a)_*[b]-R[a,b] . \end{aligned}$$

For a rational operator $R=A B^{-1}$, which is defined on ${\text {Im}}B$, we define the Nijenhuis identity as

24 $$\begin{aligned} \begin{aligned}&A_*[Aa]-[(Aa)_*,A] + AB^{-1}AB^{-1}(B_*[Ba]-[(Ba)_*,B]) \\&\quad =AB^{-1}(B_*[Aa]+A_*[Ba]-[(Aa)_*,B]-[(Ba)_*,A]) \quad \text{ for } \text{ all } \quad a \in {\mathrm F}, \end{aligned} \end{aligned}$$

where the bracket denotes the commutator of two difference operators.

### Theorem 3

If two difference operators *A* and *B* form a preHamiltonian pair, then $R=A B^{-1}$ is Nijenhuis.

### Proof

Since *A* and *B* are preHamiltonian we can write for all $a \in {\mathrm F}$

25 $$\begin{aligned} \begin{aligned} A_*[Aa]-[(Aa)_*,A]&=A(\omega _A(a,\bullet )+(Aa)_*-a_*A);\\ B_*[Ba]-[(Ba)_*,B]&=B(\omega _B(a,\bullet )+(Ba)_*-a_*B). \end{aligned} \end{aligned}$$

Hence, we see that, provided that *A* and *B* are preHamiltonians, (24) is equivalent to

26 $$\begin{aligned} \begin{aligned}&A B^{-1} \left( B\omega _A(a,\bullet )+A \omega _B(a,\bullet )-B_*[Aa]-A_*[Ba] \right. \\&\quad \left. +(Aa)_*B+(Ba)_*A-Aa_*B-Ba_*A\right) =0, \end{aligned} \end{aligned}$$

where the expression inside the parentheses is nothing else than (22). Therefore, given two preHamiltonians difference operators *A* and *B*, the ratio $AB^{-1}$ is Nijenhuis if and only if *A* and *B* form a preHamiltonian pair. $\quad \square$

Conversely, we have the following statement:

### Theorem 4

Let *R* be a Nijenhuis rational difference operator with minimal decomposition $AB^{-1}$ such that *B* is preHamiltonian. Then *A* and *B* form a preHamiltonian pair.

### Proof

Since *B* is preHamiltonian, we have for all $a \in {\mathrm F}$

27 $$\begin{aligned} B_*[Ba]-[(Ba)_*,B]=B(\omega _B(a,\bullet )+(Ba)_*-a_*B) \end{aligned}$$

Therefore, we can transform (24) into the equivalent form

28 $$\begin{aligned} \begin{aligned}&A_*[Aa]-(Aa)_*A+Aa_*A \\&\quad =AB^{-1}(B_*[Aa]+A_*[Ba]+Ba_*A\\&\qquad +Aa_*B-(Ba)_*A-(Aa)_*B-A \omega _B(a,\bullet )). \end{aligned} \end{aligned}$$

Let $CA=DB$ be the left least common multiple of the pair *A* and *B*. It is also the right least common multiple of the pair *C* and *D* since $AB^{-1}$ is minimal. By Lemma 5 (i) there exists a difference operator $\omega _A(a, \bullet )$ and thus 2–form $\omega _A$ on ${\mathrm F}$ such that

29 $$\begin{aligned} \begin{aligned}&A_*[Aa]-(Aa)_*A+Aa_*A=A \omega _A(a, \bullet ); \\&B_*[Aa]+A_*[Ba]+Ba_*A+Aa_*B-(Ba)_*A-(Aa)_*B-A \omega _B(a,\bullet )\\&\quad =B \omega _A(a,\bullet ), \end{aligned} \end{aligned}$$

which implies that *A* and *B* form a preHamiltonian pair. $\quad \square$

There is a simple algorithm to determine whether a given difference operator is preHamiltonian and to find the corresponding 2–form $\omega$. Theorem 3 provides an efficient method to check the Nijenhuis property for rational operators, which is important in the theory of integrability.

### Example 2

The operators *A* and *B* defined in (2) form a preHamiltonian pair. Thus the recursion operator for the Volterra chain (1) is Nijenhuis.

### Proof

Let $C=A+\lambda B$. According to Definition 8, we check the existence of a 2-form $\omega _C$ in (20). By direct computation, we have

$$\begin{aligned}&C_*[C a](b)-C_*[C b](a)\\&\quad = u \langle u_1 u_2 a_3 b_2+(u_1+u) u_1 a_2 b_1+(u+u_{-1})u_{-1}a_{-1}b+u_{-2} u_{-1} a_{-2} b_{-1}\\&\qquad +\lambda u_1 a_2 b_1+\lambda u_{-1}a_{-1} b\rangle _{\mathcal {P}_{a,b}}, \end{aligned}$$

where ${\mathcal {P}_{a,b}}$ stands for anti-symmetrisation with respect to $a_i$’s and $b_j$’s. We can now compute its preimage $\omega _C(a, b)$ by comparing its highest order either of *a* or *b* and we get

$$\begin{aligned} \omega _C(a, b)=u (a_1 b-a b_1)+u_{-1} ( a b_{-1} -a_{-1} b). \end{aligned}$$

It follows from Theorem 3 that the recursion operator $R=A B^{-1}$ for the Volterra chain (1) is Nijenhuis. $\quad \square$

The previous two theorems provide the interrelations between preHamiltonian pairs and Nijenhuis operators. The following theorem (analogous to its differential counterpart in [17]) gives another motivation to the definition of a preHamiltonian pair: it is a necessary condition for a rational operator $R=AB^{-1}$ to ‘generate’ an infinite commuting hierarchy.

### Theorem 5

Let *R* be a rational operator with minimal decomposition $R=AB^{-1}$. Suppose that there exist $(f^{(n)})_{n \ge 0} \in {\mathrm F}$ spanning an infinite dimensional space over ${\mathrm k}$ such that for all $n \ge 0$, $A(f^{(n)})=B(f^{(n+1)})$ and such that $[B(f^{(n)}), B(f^{(m)})]=0$ for all $n,m\ge 0$. Then *A* and *B* form a preHamiltonian pair.

### Proof

Since $[B(f^{(m)}),B(f^{(n)})]=0$ for all $m,n \ge 0$ by assumption, we have

30 $$\begin{aligned} (B_*[B(f^{(n+1)})]-(B(f^{(n+1)}))_*B)(f^{(m)})=B\left( -(f^{(m)})_*[B(f^{(n+1)})] \right) \quad \forall m,n \ge 0. \end{aligned}$$

Similarly, replacing *B* with *A* we get for all $n,m \ge 0$

31 $$\begin{aligned} (A_*[A(f^{(n)})]-(A(f^{(n)}))_*A)(f^{(m)})=A(-(f^{(m)})_*[A(f^{(n)})]). \end{aligned}$$

Let $CA=DB$ be the left least common multiple of the pair *A* and *B*. A non-zero difference operator has a finite dimensional kernel over ${\mathrm k}$, therefore one must have for all $n \ge 0$ that

32 $$\begin{aligned} D(B_*[B(f^{(n+1)})]-(B(f^{(n+1)}))_*B)=C(A_*[A(f^{(n)})]-(A(f^{(n)}))_*A). \end{aligned}$$

By minimality of the fraction $AB^{-1}$, we deduce that for all $n \ge 0$ there exists a difference operator $P^{(n)}$ such that

33 $$\begin{aligned} \begin{aligned} B_*[B(f^{(n+1)})]-(B(f^{(n+1)}))_*B&=BP^{(n)}, \\ A_*[A(f^{(n)})]-(A(f^{(n)}))_*A&=AP^{(n)}. \end{aligned} \end{aligned}$$

For all $f \in {\mathrm F}$ we can write $B(f)_*=B f_*+(D_B)_f$, where $(D_B)_f$ is defined by $(D_B)_f(g)=B_*[g](f)$ for all $g \in {\mathrm F}$. $D_B$ is a bidifference operator, i.e., $(D_B)_f$ is a difference operator and its coefficients are difference operators applied to *f*. In other words $(D_B)_f=P_M(f)S^M+\cdots P_N(f)S^N$ for all *f*, where $P_M,\ldots P_N$ are difference operators. We can find a unique pair of bidifference operators *Q* and *L* such that $\mathrm{Ord}L_f < \mathrm{Ord}B$ for all *f* and

34 $$\begin{aligned} B_*[B(f)]-(D_B)_f B=BQ_f+L_f . \end{aligned}$$

From (33) we see that $L_{f^{(n)}}=0$ for all $n \ge 1$. This implies that $L=0$ since the $f^{(n)}$ span an infinite dimensional space over ${\mathrm k}$. Therefore, for all *f*, *g*, we have

$$\begin{aligned} \begin{aligned} B_*[B(f)](g)-B_*[B(g)](f) =B Q_f(g) \end{aligned} \end{aligned}$$

implying that *B* is preHamiltonian. Finally, since for all constant $\lambda$, operator

$$\begin{aligned} R+ \lambda =(A+ \lambda B)B^{-1} \end{aligned}$$

satisfies the same hypothesis as *R*, we conclude that $A+\lambda B$ is preHamiltonian. $\quad \square$

## Towards Applications to Differential–Difference Equations

In this section we introduce some basic concepts for differential–difference equations relevant to the contents of this paper. More details on the variational difference complex and Lie derivatives can be found in [15, 36].

Let $\mathbf{u}=(u^1(n,t),\ldots ,u^N(n,t))$ be a vector function of a discrete variable $n\in {\mathbb {Z}}$ and time variable *t*, where *n* and *t* are “independent variables” and $\mathbf{u}$ will play the role of a “dependent” variable in an evolutionary differential–difference system

35 $$\begin{aligned} \mathbf{u}_t=\mathbf{f}(\mathbf{u}_p,\ldots ,\mathbf{u}_q),\qquad p\le q,\ \ p,q\in {\mathbb {Z}}. \end{aligned}$$

The Eq. (35) is an abbreviated form to encode the infinite sequence of ordinary differential systems of equations

$$\begin{aligned} \partial _t\mathbf{u}(n,t)=\mathbf{f}(\mathbf{u}(n+p,t),\ldots ,\mathbf{u}(n+q,t)),\qquad n\in {\mathbb {Z}}. \end{aligned}$$

A vector function $\mathbf{f}$ is assumed to be a locally holomorphic function in its arguments. In the majority of cases it will be a rational or polynomial function which does not depend explicitly on the variables *n*, *t*. The corresponding vector field coincides with (4). Thus there is a bijection between evolutionary derivations of ${\mathrm F}$ and differential–difference systems with $\mathbf{f}\in {\mathrm F}^N$.

### Definition 10

There are three equivalent definitions of symmetry of an evolutionary equation. We say that $\mathbf{g}\in {\mathrm F}^N$ is a symmetry of (35) if

1. $$[\mathbf{g},\mathbf{f}]=0.$$
2. $\hat{\mathbf{u}}_k=\mathbf{u}_k+\epsilon \mathbf{g}_k$ satisfy equation (35) $\mathrm{mod}\,\epsilon ^2$ whenever $\mathbf{u}$ is a solution.
3. Equation $\mathbf{u}_\tau =\mathbf{g}$ is compatible with (35).

Symmetries of an equation form a Lie subalgebra in $\mathrm{Der}\,{\mathrm F}$. The existence of an infinite dimensional commutative Lie algebra of symmetries is a characteristic property of an integrable equation and it can be taken as a definition of integrability.

Often the symmetries of integrable equations can be generated by recursion operators [2]. Roughly speaking, a recursion operator is a linear operator $R: {\mathrm F}^N\rightarrow {\mathrm F}^N$ mapping a symmetry to a new symmetry. For an evolutionary Eq. (35), it satisfies

36 $$\begin{aligned} R_t=R_*[\mathbf{f}]=[ \mathbf{f}_*,\ R] . \end{aligned}$$

Recursion operators for nonlinear integrable equations are often Nijenhuis operators. Therefore, if the Nijenhuis operator *R* is a recursion operator of (35), the operator *R* is also a recursion operator for each of the evolutionary equations in the hierarchy $\mathbf{u}_t=R^k(\mathbf{f})$, where $k=0,1,2,\ldots \ .$

Nijenhuis operators are closely related to Hamiltonian and symplectic operators. The general framework in the context of difference variational complex and Lie derivatives can be found in [15, 36]. Here we recall the basic definitions related to Hamiltonian systems.

For any element $a\in {\mathrm F}$, we define an equivalent class (or a functional) $\int \! a$ by saying that two elements $a,b\in {\mathrm F}$ are equivalent if $a-b\in \text{ Im }({\mathcal S}-1)$. The space of functionals is denoted by ${\mathrm F}'$.

For any functional $\int \!\!f\in {\mathrm F}'$ (simply written $f\in {\mathrm F}'$ without confusion), we define its difference variational derivative (Euler operator) denoted by $\delta _{\mathbf{u}} f \in {\mathrm F}^N$ (here we identify the dual space with itself) as

$$\begin{aligned} \delta _{\mathbf{u}} f=\left( \delta _{u^1} f, \ldots , \delta _{u^N} f \right) ^{tr},\qquad \delta _{u^l} f=\sum _{i\in {\mathbb {Z}}} {\mathcal S}^{-i} \frac{\partial f}{\partial u_{i}^l}= \frac{\partial }{\partial u^l}\left( \sum _{i\in {\mathbb {Z}}} {\mathcal S}^{-i} f \right) . \end{aligned}$$

### Definition 11

An evolutionary Eq. (35) is said to be a Hamiltonian equation if there exists a Hamiltonian operator *H* and a Hamiltonian $\int g\in {\mathrm F}'$ such that $\mathbf{u}_t=H \delta _{\mathbf{u}} \int g.$

This is the same to say that the evolutionary vector field $\mathbf{f}$ is a Hamiltonian vector field and thus the Hamiltonian operator is invariant along it, that is,

37 $$\begin{aligned} H_t=H_*[\mathbf{f}]=\mathbf{f}_* H+H \mathbf{f}_*^{\dagger } . \end{aligned}$$

Nijenhuis recursion operators for some integrable difference equations, e.g., the Narita-Itoh-Bogoyavlensky lattice [37], are no longer weakly nonlocal, but rational difference operators of the form $R=AB^{-1}$. The following statement tells us how operators *A* and *B* are related to a given equation.

### Theorem 6

If a rational difference operator *R* with minimal decomposition $AB^{-1}$ is a recursion operator for Eq. (35), then there exists a difference operator *P* such that

38 $$\begin{aligned} A_*[\mathbf{f}]=\mathbf{f}_*A+AP, \qquad B_*[\mathbf{f}]=\mathbf{f}_*B+BP . \end{aligned}$$

### Proof

To say that $AB^{-1}$ is a minimal decomposition of *R* means that *A* and *B* are right coprime. Let *C* and *D* be two left coprime matrix operators with *C* regular such that $CA=DB$. Such a pair exists by Lemma 5. Since $R=AB^{-1}$ is a recursion operator of (35), substituting it into (36) we have

$$\begin{aligned} R_t=R_*[\mathbf{f}]=A_*[\mathbf{f}]B^{-1}-A B^{-1} B_*[\mathbf{f}] B^{-1}= \mathbf{f}_* A B^{-1}-A B^{-1}\mathbf{f}_* , \end{aligned}$$

that is,

39 $$\begin{aligned} \left( A_*[\mathbf{f}]-\mathbf{f}_*A \right) =A B^{-1}\left( B_*[\mathbf{f}]-\mathbf{f}_*B\right) . \end{aligned}$$

We rewrite (39) as

$$\begin{aligned} C \left( A_*[\mathbf{f}]-\mathbf{f}_*A \right) =D \left( B_*[\mathbf{f}]-\mathbf{f}_*B\right) . \end{aligned}$$

By Lemma 5 there exists an operator *P* such that

$$\begin{aligned}&A_*[\mathbf{f}]-\mathbf{f}_*A=A P,\qquad B_*[\mathbf{f}]-\mathbf{f}_*B=B P. \end{aligned}$$

Thus the operators *A* and *B* satisfy the same relation (38). $\quad \square$

Comparing to (37), for Hamiltonian operators, we have $P=\mathbf{f}_*^{\dagger }$. Conversely, it can be easy to show that

### Proposition 10

For an Eq. (35) if there exist two operators *A* and *B* satisfying (38), then $R=AB^{-1}$ is a recursion operator for the equation.

### Proof

By direct computation, we have

$$\begin{aligned} R_t= & {} A_*[\mathbf{f}]B^{-1}-A B^{-1} B_*[\mathbf{f}] B^{-1}\\= & {} (\mathbf{f}_*A+A P)B^{-1}-A B^{-1} (\mathbf{f}_*B+B P)B^{-1}=\mathbf{f}_*R-R\mathbf{f}_* \end{aligned}$$

satisfying (36). Thus $R=A B^{-1}$ is a recursion operator. $\quad \square$

This proposition has been used in [38] in constructing recursion operators for integrable noncommutative ODEs.

### Example 3

For the operators *A* and *B* defined in (2) of the Volterra chain (1), the difference operator *P* in Theorem 6 is $P=(1+{\mathcal S}^{-1})u (1-{\mathcal S})$.

In what follows, we give the conditions for a rational recursion operator $R=AB^{-1}$ to generate infinitely many local commuting symmetries. We first prove the following lemma:

### Lemma 3

Assume that *B* is a preHamiltonian operator $R=AB^{-1}$ with minimal decomposition is a recursion operator for $\mathbf{u}_t=B(\mathbf{g})$, where $\mathbf{g}\in {\mathrm F}^N$. Then $[B(\mathbf{g}),A(\mathbf{g})]=0$.

In particular, if there exists $\mathbf{h}\in {\mathrm F}^N$ such that *R* is a recursion operator for $\mathbf{u}_t=B(\mathbf{h})$ and $[B(\mathbf{g}),A(\mathbf{h})]=0$, then $[A(\mathbf{g}),B(\mathbf{h})]=0$.

### Proof

We know that *B* is preHamiltonian. So for any $\mathbf{a}\in {\mathrm F}^N$, we have

40 $$\begin{aligned} B_*[B\mathbf{a}]-(B\mathbf{a})_*B&=B(\omega _B(\mathbf{a},\bullet )-\mathbf{a}_*B). \end{aligned}$$

From Theorem 6, it follows, when $\mathbf{a}=\mathbf{g}$ or $\mathbf{a}=\mathbf{h}$, that

41 $$\begin{aligned} A_*[B\mathbf{a}]-(B\mathbf{a})_*A&=A(\omega _B(\mathbf{a},\bullet )-\mathbf{a}_*B). \end{aligned}$$

Using (41) for $\mathbf{a}=\mathbf{g}$, we get

$$\begin{aligned} {[}B(\mathbf{g}),A(\mathbf{g})]= A_*[B(\mathbf{g})](\mathbf{g})+A{\mathbf{g}}_*[B(\mathbf{g})]-(B\mathbf{g})_*[A \mathbf{g}]=A(\omega _B(\mathbf{g},\mathbf{g}))=0. \end{aligned}$$

If there exists $\mathbf{h}\in {\mathrm F}^N$ such that *R* is a recursion operator for $\mathbf{u}_t=B(\mathbf{h})$ then from the former we deduce that

42 $$\begin{aligned} {[}B(\mathbf{g}+\mathbf{h}),A(\mathbf{g}+\mathbf{h})]=[B(\mathbf{g}),A(\mathbf{g})]=[B(\mathbf{h}),A(\mathbf{h})]=0. \end{aligned}$$

Hence $$[B(\mathbf{h}),A(\mathbf{g})]=-[B(\mathbf{g}),A(\mathbf{h})]$$. $$\quad \square $$

### Proposition 11

Assume that *A* and *B* form a preHamiltonian pair and $R=AB^{-1}$ is a recursion operator for $\mathbf{u}_t=B(\mathbf{g}^{(0)})$, where $\mathbf{g}^{(0)}\in {\mathrm F}^N$. If there exists $\mathbf{g}^{(n)}\in {\mathrm F}^N$ such that $A(\mathbf{g}^{(n)})=B(\mathbf{g}^{(n+1)})$ for all $n \ge 0$, then $[B(\mathbf{g}^{(n)}),B(\mathbf{g}^{(m)})]=0$ for all $n,m \ge 0$.

### Proof

We can assume that $AB^{-1}$ is a minimal decomposition of *R*. Indeed, if not we write $A=A_0C$ and $B=B_0C$ where $R=A_0B_0^{-1}$ is minimal and replace $\mathbf{g}^{(n)}$ by $C(\mathbf{g}^{(n)})$. By Theorem 3, we know that *R* is Nijenhuis and thus it is a recursion operator for all $B(\mathbf{g}^{(n)})$, $n\ge 0$. We proceed the proof by induction on $|n-m|$. If $n=m$ there is nothing to prove. If $|n-m|=1$, we deduce $[B(\mathbf{g}^{(n)}),B(\mathbf{g}^{(n+1)})]=0$ as a direct application of Lemma 3 since $B(\mathbf{g}^{(n+1)})=A(\mathbf{g}^{(n)})$ for all $n \ge 0$. Suppose that $[B(\mathbf{g}^{(n)}),B(\mathbf{g}^{(m)})]=0$ for all $n,m \ge 0$ such that $|n-m| \le N$, which implies $[B(\mathbf{g}^{(n+N)},B(\mathbf{g}^{(n+1)})]=0$. Hence by Lemma 3, we have $[B(\mathbf{g}^{(n+N+1)},B(\mathbf{g}^{(n)})]=0$.

$$\square $$

## Rational Recursion Operator for Adler–Postnikov Equation

In this section, we construct a recursion operator of system

43 $$\begin{aligned} u_t=u^2(u_2 u_1-u_{-1} u_{-2})-u (u_1-u_{-1}):=f \end{aligned}$$

from its Lax representation and show that it is Nijenhuis and generates local commuting symmetries. In general, it is not easy to construct a recursion operator for a given integrable equation although the explicit formula is given. The difficulty lies in how to determine the starting terms of *R*, i.e., the order of the operator, and how to construct its nonlocal terms. Many papers are devoted to this subject, see [5, 39, 40]. If the Lax representation of the equation is known, there is an amazingly simple approach to construct a recursion operator proposed in [41]. The idea in [41] can be developed for the Lax pairs that are invariant under the reduction groups, which applies for both differential and differential–difference equations [7, 37].

The Eq. (43) first appeared in [18], where the authors presented its scalar Lax representation. We rewrite it in the matrix form as follows:

44 $$\begin{aligned}&L={\mathcal S}-\mathbf{U}(\lambda )={\mathcal S}-\lambda \mathbf{U}^{(1)}-\mathbf{U}^{(0)}={\mathcal S}- \left( {\begin{array}{*{5}c} 0 &{}1 &{}0\\ 0 &{}0 &{}1\\ \lambda &{} -\frac{1}{u} &{} \frac{\lambda }{u} \end{array}}\right) \end{aligned}$$

45 $$\begin{aligned}&M=D_t-\mathbf{V}(\lambda )=D_t+ \left( {\begin{array}{*{5}c} -\frac{1}{\lambda ^2}+u_{-1} &{}\frac{1}{\lambda }(1-u_{-1} u_{-2}) &{}u_{-1}u_{-2}-\frac{u_{-1}}{\lambda ^2}\\ \lambda u u_{-1}-\frac{u}{\lambda } &{}u-u_{-1} &{}\lambda u_{-1}-\frac{u u_{-1}}{\lambda }\\ \lambda ^2 u- u u_1 &{} \lambda (u u_1-1) &{} \lambda ^2-u \end{array}}\right) , \end{aligned}$$

where $\lambda$ is a spectral parameter. The commutativity of the above operators leads to the zero curvature condition

46 $$\begin{aligned} \mathbf{U}(\lambda )_t={\mathcal S}\mathbf{V}(\lambda ){\mathcal S}^{-1} \mathbf{U}(\lambda )-\mathbf{U}(\lambda ) \mathbf{V}(\lambda ) \end{aligned}$$

and subsequently it leads to the system (43). The system (43) defines a derivation $X_f\in {\mathcal {A}}_1$ of ${\mathcal {R}}$ with $f=u^2(u_2 u_1-u_{-1} u_{-2})-u (u_1-u_{-1})$. The representation (44), (45) is invariant with respect to the transformations:

47 $$\begin{aligned} {\mathcal S}\mathbf{V}(\lambda ){\mathcal S}^{-1}=-J {\mathcal {T}}\mathbf{V}(\lambda ^{-1}) {\mathcal {T}}J,\qquad \mathbf{U}^{-1}(\lambda )= J {\mathcal {T}}\mathbf{U}(\lambda ^{-1}) {\mathcal {T}}J \end{aligned}$$

and

48 $$\begin{aligned} \mathbf{V}(\lambda )=H \mathbf{V}(-\lambda ) H,\qquad \mathbf{U}(\lambda )= -H\mathbf{U}(-\lambda )H, \end{aligned}$$

where

$$\begin{aligned} J=\left( \begin{array}{r@{\quad }r@{\quad }r} 0&{}0&{}1\\ 0&{}1&{}0\\ 1&{}0&{}0 \end{array}\right) ,\qquad H=\left( \begin{array}{r@{\quad }r@{\quad }r} 1&{}0&{}0\\ 0&{}-1&{}0\\ 0&{}0&{}1 \end{array}\right) . \end{aligned}$$

The transformation (47) reflects the symmetry ${\mathcal {T}}(f)=-f$ of the Eq. (43).

For a given matrix $\mathbf{U}$, we can build up a hierarchy of nonlinear systems by choosing different matrices $\mathbf{V}$ with the degree of $\lambda$ from $-2l$ to 2*l*. The way to construct a recursion operator directly from a Lax representation is to relate the different operators $\mathbf{V}$ using ansatz

$$\begin{aligned} {\bar{\mathbf{V}}}=(\lambda ^{-2}+\lambda ^2) \mathbf{V}+\mathbf{W}\end{aligned}$$

and then to find the relation between the two flows corresponding to $\bar{\mathbf{V}}$ and $\mathbf{V}$. The multiplier $\lambda ^{-2}+\lambda ^2$ is the automorphic function of the group generated by the transformations $\lambda \mapsto \lambda ^{-1}$ and $\lambda \mapsto -\lambda$. Here $\mathbf{W}$ is the remainder and we assume that it has the same symmetry as $\mathbf{V}$:

49 $$\begin{aligned} \mathbf{W}=\sum _{j=-2}^{2} {\lambda }^{j} \mathbf{W}^{(j)} , \end{aligned}$$

where $\mathbf{W}^{(j)}$ are $3\times 3$ matrices of the following form [invariant under (48)]

$$\begin{aligned}&\mathbf{W}^{(2)}=\left( {\begin{array}{*{5}c} a&{}0&{}b\\ 0&{}c&{}0\\ d&{}0&{}e \end{array}}\right) ; \quad \mathbf{W}^{(0)}=\left( {\begin{array}{*{5}c} a_0&{}0&{}b_0\\ 0&{}c_0&{}0\\ d_0&{}0&{}e_0 \end{array}}\right) ;\quad \mathbf{W}^{(-2)}=\left( {\begin{array}{*{5}c} a_{-}&{}0&{}b_{-}\\ 0&{}c_{-}&{}0\\ d_{-}&{}0&{}e_{-} \end{array}}\right) ;\\&\mathbf{W}^{(1)}=\left( {\begin{array}{*{5}c} 0&{}r&{}0\\ s&{}0&{}p\\ 0&{}q&{}0 \end{array}}\right) ; \quad \mathbf{W}^{(-1)}=\left( {\begin{array}{*{5}c} 0&{}r_{-}&{}0\\ s_{-}&{}0&{}p_{-}\\ 0&{}q_{-}&{}0 \end{array}}\right) \end{aligned}$$

and since $\mathbf{W}$ is invariant under (47), they satisfy

$$\begin{aligned} {\mathcal S}(\mathbf{W}^{(2)})= & {} -J {\mathcal {T}}(\mathbf{W}^{(-2)}) J; \quad {\mathcal S}(\mathbf{W}^{(1)})=-J {\mathcal {T}}(\mathbf{W}^{(-1)}) J; \\ {\mathcal S}(\mathbf{W}^{(0)})= & {} -J {\mathcal {T}}(\mathbf{W}^{(0)}) J. \end{aligned}$$

The zero curvature condition leads to

50 $$\begin{aligned} \begin{array}{l} \mathbf{U}_{\tau }=\left( \frac{1}{\lambda ^2}+\lambda ^2\right) \mathbf{U}_t+{\mathcal S}(\mathbf{W}) \mathbf{U}-\mathbf{U}\mathbf{W}. \end{array} \end{aligned}$$

Substituting the ansatz (49) into (50) and collecting the coefficient of powers of ${\lambda }$, we obtain six matrix equations for $\mathbf{W}^{(j)}, j=-2, \ldots , 2$. For example, the equation corresponding to linear terms of ${\lambda }$ is

51 $$\begin{aligned} \mathbf{U}^{(1)}_{\tau }={\mathcal S}(\mathbf{W}^{(1)}) \mathbf{U}^{(0)}+{\mathcal S}(\mathbf{W}^{(0)}) \mathbf{U}^{(1)}- \mathbf{U}^{(1)} \mathbf{W}^{(0)}-\mathbf{U}^{(0)} \mathbf{W}^{(1)}. \end{aligned}$$

Through them we are able to determine the entries of matrices $\mathbf{W}^{(j)}$ and we finally get

52 $$\begin{aligned}&c_{-}=({\mathcal S}^2-1)^{-1}\frac{u_t}{u};\nonumber \\&b_0={\mathcal S}^{-1}u ({\mathcal S}u-u {\mathcal S}^{-1})^{-1} \left( u({\mathcal S}-{\mathcal S}^{-2})u ({\mathcal S}^2+{\mathcal S}+1)+{\mathcal S}^2-{\mathcal S}\right) c_{-};\nonumber \\&e_0=({\mathcal S}^2+{\mathcal S}+1)^{-1} \left( -\left( {\mathcal S}\frac{1}{u} {\mathcal S}+\frac{1}{u} {\mathcal S}+{\mathcal S}\frac{1}{u}\right) {\mathcal S}b_0+({\mathcal S}+1)\frac{1}{u}({\mathcal S}^2-{\mathcal S})c_{-}\right) ;\nonumber \\&u_{\tau }=u^2({\mathcal S}^3-1)b_0-u (u {\mathcal S}-{\mathcal S}^{-1} u) ({\mathcal S}^2+{\mathcal S}+1) c_{-}+u ({\mathcal S}-1)e_0. \end{aligned}$$

Note that

$$\begin{aligned} {\mathcal S}\frac{1}{u} {\mathcal S}u+\frac{1}{u} {\mathcal S}u+{\mathcal S}=({\mathcal S}+1) \frac{1}{u} ({\mathcal S}u-u {\mathcal S}^{-1})+{\mathcal S}^{-1}+1+{\mathcal S}. \end{aligned}$$

We simplify the above expression of $e_0$. It becomes

$$\begin{aligned} e_0= & {} ({\mathcal S}^{-2}-1)u ({\mathcal S}^2+{\mathcal S}+1) c_{-} -{\mathcal S}^{-1} ({\mathcal S}u-u {\mathcal S}^{-1})^{-1} \\&\quad \left( u({\mathcal S}-{\mathcal S}^{-2})u ({\mathcal S}^2+{\mathcal S}+1)+{\mathcal S}^2-{\mathcal S}\right) c_{-} \end{aligned}$$

Substituting $c_{-}, b_0$ and $e_0$ into (52), we obtain the relation between two symmetry flows $u_t$ and $u_{\tau }$. Thus we obtain the following statement:

### Proposition 12

A recursion operator for Eq. (43) is

53 $$\begin{aligned}&R=u\left( u ({\mathcal S}^2\!-\!{\mathcal S}^{-1}\!) u +{\mathcal S}^{-1}\!\!\!-\!1\right) ({\mathcal S}u\!-\!u{\mathcal S}^{-1}\!)^{-1}\nonumber \\&\quad \left( u ({\mathcal S}\!-\!{\mathcal S}^{-2}\!)u ({\mathcal S}^2+{\mathcal S}+1)+{\mathcal S}^2\!-\!{\mathcal S}\right) ({\mathcal S}^2\!-\!1)^{-1}\! \frac{1}{u}\nonumber \\&\quad +u(2 {\mathcal S}^{-1} u-{\mathcal S}^{-2} u-{\mathcal S}u+u-u{\mathcal S}) ({\mathcal S}^2+{\mathcal S}+1)({\mathcal S}^2-1)^{-1}\frac{1}{u}. \end{aligned}$$

We represent *R* as

$$\begin{aligned} R=R^{(3)}+R^{(1)}+R^{(-1)}, \end{aligned}$$

where

$$\begin{aligned}&R^{(3)}=u^2 ({\mathcal S}^3-1){\mathcal S}^{-1}u ({\mathcal S}u-u{\mathcal S}^{-1})^{-1} u ({\mathcal S}-{\mathcal S}^{-2})u ({\mathcal S}^2+{\mathcal S}+1) ({\mathcal S}^2-1)^{-1} \frac{1}{u};\\&R^{(-1)}=u ({\mathcal S}^{-1}-1) ({\mathcal S}u-u {\mathcal S}^{-1})^{-1} ({\mathcal S}^{-1}+1)^{-1} \frac{1}{u}. \end{aligned}$$

Note that $R^{(3)}$ is a recursion operator for $u_t=u^2(u_1 u_2-u_{-1} u_{-2})$ [37] and that $R^{(-1)}$ is the inverse recursion operator for the Volterra chain $u_t=u (u_1-u_{-1})$ [20].

The recursion operator (53) is not weakly nonlocal. We now rewrite it as a rational difference operator. It is convenient to first write *R* as

54 $$\begin{aligned} R=\left( Q\Delta ^{-1}C+P\right) ({\mathcal S}^2-1)^{-1} \frac{1}{u}, \end{aligned}$$

where

55 $$\begin{aligned}&Q=u\left( uu_1-1+(1-uu_{-1})S^{-1}\right) ; \ \ \Delta ={\mathcal S}u -u {\mathcal S}^{-1}; \end{aligned}$$

56 $$\begin{aligned}&C=w_2{\mathcal S}^2-w_{-1} {\mathcal S}, \quad w=1-u_{-1} u_1; \end{aligned}$$

57 $$\begin{aligned}&P=\left( u^2{\mathcal S}u ({\mathcal S}-{\mathcal S}^{-2})u\right. \nonumber \\&\quad \left. +Q u_{-1}+ u(2 {\mathcal S}^{-1} u-{\mathcal S}^{-2} u-{\mathcal S}u+u-u{\mathcal S})\right) ({\mathcal S}^2+{\mathcal S}+1)\nonumber \\&\quad +u^2 {\mathcal S}({\mathcal S}^2-{\mathcal S})+Q(u_{-1} {\mathcal S}^{-1}+u_2 {\mathcal S})=\tilde{P}(S+1)+p; \end{aligned}$$

where ${\tilde{P}}$ is a difference operator and $p=u(u_2+2u_1-2u_{-1}-u_{-2})$.

### Lemma 4

The recursion operator *R* given by (54) can be factorized as $R=AB^{-1}$ with

58 $$\begin{aligned} B=u({\mathcal S}-{\mathcal S}^{-1})({\mathcal S}\alpha + \beta +{\mathcal S}^{-1}\gamma ), \end{aligned}$$

where

$$\begin{aligned}&\alpha =u_{-1}uw_{-1}w-u_{-1}u_1w_1w_2;\quad \beta =u^2w^2-u_{-1}u_1w_{-2}w_2;\\&\gamma =u_{1}uw_{1}w-u_{-1}u_1w_{-1}w_{-2}; \quad w=1-u_{-1} u_1. \end{aligned}$$

and

59 $$\begin{aligned} A=Q \left( \frac{1}{u}w_1 \alpha _1 {\mathcal S}+\frac{1}{u_1} w \gamma \right) +P{\mathcal S}^{-1} \left( S \alpha + \beta + S^{-1}\gamma \right) . \end{aligned}$$

### Proof

To find *A* and *B* for (54) we need to rewrite $\Delta ^{-1}C$ as a right fraction. It turns out that

$$\begin{aligned} C {\mathcal S}^{-1}\left( {\mathcal S}\alpha + \beta + {\mathcal S}^{-1}\gamma \right) = \Delta \left( \frac{1}{u}w_1 \alpha _1 {\mathcal S}+\frac{1}{u_1} w \gamma \right) , \end{aligned}$$

from which we can find that $\alpha , \beta$ and $\gamma$ as stated is a solution. Then $A=R B$ by definition as given in the statement. $\quad \square$

The authors in [42] showed that the recursion operators derived from certain Lax representations under certain boundary conditions are Nijenhuis once every step is uniquely determined. Here we prove the Nijenhuis property using the results in Sect. 3.

### Theorem 7

The operators *A* and *B* defined by (59) and (58) are compatible preHamiltonian operators. In particular, the recursion operator *R* for Eq. (43) given by (53) is Nijenhuis.

### Proof

We know from Lemma 4 that $R=AB^{-1}$. To prove that it is Nijenhuis, we only need to show operators *A* and *B* form a preHamiltonian pair following from Theorem 3.

Let $I=A+\lambda B$. For any $a, b\in {\mathrm F}$ and constant $\lambda$, we use computer algebra package Maple to compute $e^{(0)}=I_*[Ia](b)-I_*[Ib](a)$, which is linear in *a* and its shifts. We take the coefficient of the highest order term $a_k$ (here $k=11$) in $e^{(0)}$ and denote it by $v^{(0)}$. Notice that the highest order term in *I* is $u^2 u_1 u_2 {\mathcal S}^4 \alpha$. We set $\omega ^{(0)}=\frac{1}{\alpha }{\mathcal S}^{-4}(\frac{v^{(0)} a_k}{u^2 u_1 u_2 })$. We then compute $e^{(1)}=e^{(0)}-I(\omega ^{(0)})$ and repeat the procedure. Finally we get $e^{(11)}=0$ after $n=11$ steps implying *I* is preHamiltonian. $\quad \square$

Since the operator *R* is not weakly nonlocal, the results on the locality of symmetries generated by *R* in [7] are no longer valid. In the rest of this section, we are going to show that *R* generates infinitely many commuting symmetries of (43) starting from the equation itself.

### Proposition 13

Let *h* be a difference polynomial such that *R* is a recursion operator for $u_t=h$. Then *h* lies in the image of *B*. More precisely $h=B(x)$ for some $x \in {\mathrm F}$ and *A*(*x*) is a difference polynomial. Moreover, *R* is a recursion operator for $u_t=A(x)$.

We will break the proof of this proposition in two parts using (54). First we will prove that $h=u(g_2-g)$ for some difference polynomial *g*. Second we will show that $C(g)=\Delta (k)$ for some difference polynomial *k*. We begin with proving a few lemmas. To improve the readability, we put them in “Appendix B”. We now write the proof for Proposition 13 using these lemmas.

### Proof

By Lemma 8, we know that $h=u(g_2-g)$ for some difference polynomial *h*. By Lemmas 9 and 10, for some constant $\lambda \in {\mathrm k}$ we get that

$$\begin{aligned} C(g)_*u(S^2-1)-C(g)(S^2-S) \equiv \lambda C. \end{aligned}$$

Since *g* is a difference polynomial, the constant term in $C(g)_*u(S^2-1)-C(g)(S^2-S)-\lambda C$ is $\lambda (S-S^2)$. This constant term must be divisible on the left by $\Delta$, which implies $\lambda =0$. Moreover, we can divide the congruence relation by $(S^2-S)$ on the right since $\Delta$ has a trivial kernel:

$$\begin{aligned} C(g)_*u(1+S^{-1}) \equiv C(g). \end{aligned}$$

After applying Lemma 11 we deduce that $C(g)=\Delta (k)$ for some difference polynomial *k*.

Let *M* be a generator of the right ideal $C {\mathcal {R}}\cap \Delta {\mathcal {R}}$ in ${\mathcal {R}}$. This means that $M=CE=\Delta D$ for some pair of right coprime difference operators *D* and *E*. By Lemma 1, there exists $x \in {\mathrm F}$ such that $g=E(x)$ and $k=D(x)$. Since $B=u(S^2-1)E$, we conclude that $h=B(x)$. Finally, $A=QD+PE$, hence $A(x)=P(g)+Q(k)$ is a difference polynomial. *R* is a recursion operator for $u_t=A(x)$ since *R* is Nijenhuis following from Theorem 7. $\quad \square$

### Theorem 8

There exists a sequence $g^{(2)},g^{(4)},g^{(6)},\ldots$ in ${\mathrm F}$ such that

1. $$u^2(u_1u_2-u_{-1}u_{-2})-u(u_1-u_{-1})=B(g^{(2)})$$;
2. $$A(g^{(2n)})=B(g^{(2n+2)}) \text { for all }n \ge 1$$;
3. $B(g^{(2n)})$ is a difference polynomial for all $n \ge 1$;
4. $$ [B(g^{(2n)}),B(g^{(2m)})]=0$$ for all $$n,m \ge 1$$;
5. The order of $B(g^{(2n)})$ is $(-2n,2n)$;
6. *R* is a recursion operator for all the $u_t=B(g^{(2n)})$.

Finally, let $V=\text {Span}_{{\mathrm k}} \{ B(g^{(2n)}) | n \ge 1 \}$. If $f \in {\mathrm F}$ commutes with some element $h \in V$, then $f \in V$.

### Proof

We already know that *R* is a recursion operator for (43), hence by Proposition 13 there exists $g^{(2)} \in {\mathrm F}$ such that statement (1) is satisfied and $A(g^{(2)})$ is a difference polynomial. Since *R* is Nijenhuis (following from Theorem 7) it must be a recursion operator for $u_t=A(g^{(2)})$ as well. Using Proposition 13 a second time we find $g^{(4)}\in {\mathrm F}$ such that $B(g^{(4)})=A(g^{(2)})$ and $A(g^{(4)})$ is a difference polynomial. Iterating this argument we prove the statements (2), (3) and (6). Statement (5) is obvious and statement (4) follows from Proposition 11 and Theorem 7. Finally, if $f \in {\mathrm F}$ commutes with $h \in V$, let us sketch the proof of how to show that $f \in V$. If (*M*, *N*) is the order of *f* and $N>0$ it is not hard to prove from the equation

60 $$\begin{aligned} X_h(f_*)=[h_*,f_*]+X_f(h_*). \end{aligned}$$

Note that the leading term of $f_*$ is up to multiplication by a constant the leading term of $B(g^{(2k)})_*$ for some $k \ge 2$. Similarly, if $M<0$, one sees that the negative leading term of $f_*$ is up to multiplication by a constant the negative leading term of $B(g^{(2l)})_*$ for some $l \ge 2$. We conclude by induction on the total order of *f*, after checking that the only *f* commuting with an element of *V* and which depend either on $u,\ldots ,u_N$ or on $u_{-N},\ldots ,0$ for $N \ge 0$ is $f=0$. $\quad \square$

### Remark 3

Note that $g^{(2)}=\frac{u_{-1}uu_1w_{-1}ww_1}{\alpha \gamma }$ is in ${\mathrm F}$ but is not a difference polynomial.

### Remark 4

Let ${\mathcal {T}}$ be the automorphism of $\mathrm {K}$ defined in Sect. 2. Then we have ${\mathcal {T}}A {\mathcal {T}}=-A$ and ${\mathcal {T}}B {\mathcal {T}}=-B.$ This implies that ${\mathcal {T}}(B(g^{(2n)}))=-B(g^{(2n)})$ and ${\mathcal {T}}(g^{(2n)})=g^{(2n)}$ for all $n \ge 1$.

## On Inverse Nijenhuis Recursion Operators

In [20], the authors listed integrable differential–difference equations with their algebraic properties. For some systems, they presented both recursion operators and their inverse in weakly nonlocal form. In this section, we’ll explain the (non)existence of weakly nonlocal inverse recursion operators and how to work out the nonlocal terms based on Theorem 1 and its corollaries in Sect. 2.3 using examples in [20].

We select four examples: in Sect. 6.1, we show the nonexistence of weakly nonlocal inverse recursion operator for the Toda lattice; in Sect. 6.2, we show the existence of weakly nonlocal inverse recursion operator with only one nonlocal term for a relativistic Toda system; in Sect. 6.3, we deal with a recursion operator with two nonlocal terms; for our last example, we demonstrate that the inverse operator *R* itself is not weakly nonlocal, but that of $R-\mathrm{id}$ is!

### The Toda lattice

The Toda equation [43] is given by

$$\begin{aligned} q_{tt}=\exp (q_{1}-q)-\exp (q-q_{-1}). \end{aligned}$$

In the Manakov-Flaschka coordinates [44, 45] defined by $u=\exp (q_1-q),\ v=q_{t}$, it can be rewritten as two-component evolution system:

61 $$\begin{aligned} \left\{ {{\begin{array}{*{5}c} u_t= u(v_{1}-v)\\ v_t= u-u_{-1} \end{array}} } \right. , \end{aligned}$$

which admits two compatible Hamiltonian local structures

$$\begin{aligned} H_1=\left( {\begin{array}{*{5}c} 0&{}u({\mathcal S}-1)\\ (1-{\mathcal S}^{-1})u&{}0 \end{array}}\right) , \quad H_2=\left( {\begin{array}{*{5}c} u({\mathcal S}-{\mathcal S}^{-1})u&{}u({\mathcal S}-1)v\\ v(1-{\mathcal S}^{-1})u&{}u{\mathcal S}-{\mathcal S}^{-1}u \end{array}}\right) . \end{aligned}$$

It is clear to see that $\mathrm{Ord} H_1=2$ and that the kernel of $H_1$ is spanned by $$\left( {\begin{array}{*{5}c} \frac{1}{u} \\ 0 \end{array}} \right) $$ and $$\left( {\begin{array}{*{5}c} 0 \\ 1 \end{array}} \right) $$. One can check that the kernel of $H_2$ is spanned by $$\left( {\begin{array}{*{5}c} \frac{1}{u} \\ 0 \end{array}} \right) $$. In other words, $H_1$ and $H_2$ have a common right divisor *C* of the total order being 1 and can be written as $H_1=BC$ and $H_2=AC$, where $\mathrm{Ord} B=1$ and $\mathrm{Ord} A=3$, that is,

$$\begin{aligned}&H_1=BC=\left( {\begin{array}{*{5}c} 0&{} u (1-{\mathcal S})\\ 1&{}0 \end{array}}\right) \left( {\begin{array}{*{5}c} (1-{\mathcal S}^{-1})u&{}0\\ 0&{}1 \end{array}}\right) ;\\&H_2=AC=\left( {\begin{array}{*{5}c} u({\mathcal S}+1)&{}u({\mathcal S}-1)v\\ v&{}u{\mathcal S}-{\mathcal S}^{-1}u \end{array}}\right) \left( {\begin{array}{*{5}c} (1-{\mathcal S}^{-1})u&{}0\\ 0&{}1 \end{array}}\right) . \end{aligned}$$

Thus *B* has full kernel and *A* has trivial kernel. Thus the recursion operator

$$\begin{aligned} R=H_2 H_1^{-1}=AB^{-1} \end{aligned}$$

is weakly nonlocal but $BA^{-1}$ is not. Indeed,

$$\begin{aligned}&R=\left( {\begin{array}{*{5}c} v_1&{} u{\mathcal S}+u \\ 1+{\mathcal S}^{-1}&{}v \end{array}}\right) +\left( {\begin{array}{*{5}c} u(v_1-v) \\ u-u_{-1} \end{array}}\right) ({\mathcal S}-1)^{-1} \left( {\begin{array}{*{5}c} \frac{1}{u}&0 \end{array}}\right) . \end{aligned}$$

### A relativistic Toda system 

The relativistic Toda system [46] is given by

$$\begin{aligned} q_{tt}=q_{t}q_{-1t}\frac{\exp (q_{-1}-q)}{1+\exp (q_{-1}-q)}-q_{t}q_{1t}\frac{ \exp (q-q_{1})}{1+\exp (q-q_{1})} . \end{aligned}$$

Introducing the dependent variables as follows [47]:

$$\begin{aligned} u=\frac{q_{t} \exp (q-q_1)}{1+\exp (q-q_1)},\qquad v=\frac{q_{t}}{1+\exp (q-q_1)}, \end{aligned}$$

then the equation can be written as

$$\begin{aligned} \left\{ {{\begin{array}{*{5}c} u_t= u(u_{-1}-u_1+v-v_1)\\ v_t= v(u_{-1}-u). \end{array}} } \right. \end{aligned}$$

It admits two compatible Hamiltonian local structures

$$\begin{aligned} H_1=\left( {\begin{array}{*{5}c} 0&{}u(1-{\mathcal S})\\ ({\mathcal S}^{-1}-1)u&{}u{\mathcal S}-{\mathcal S}^{-1}u \end{array}}\right) , \quad H_2=\left( {\begin{array}{*{5}c} u({\mathcal S}^{-1}-{\mathcal S})u&{}u(1-{\mathcal S})v\\ v({\mathcal S}^{-1}-1)u&{}0 \end{array}}\right) . \end{aligned}$$

It is clear to see that $\mathrm{Ord} H_1=2$ and that the kernel of $H_1$ is spanned by $$\left( {\begin{array}{*{5}c} \frac{1}{u} \\ 0 \end{array}} \right) $$ and $$\left( {\begin{array}{*{5}c} 1 \\ 1 \end{array}} \right) $$. Similarly $\mathrm{Ord} H_2=2$ and the kernel of $H_2$ is spanned by $$\left( {\begin{array}{*{5}c} \frac{1}{u} \\ 0 \end{array}} \right) $$ and $$\left( {\begin{array}{*{5}c} 0 \\ \frac{1}{v} \end{array}} \right) $$. In other words, $H_1$ and $H_2$ have a common right divisor $\mathrm{Ord} C=1$ and can be written as

$$\begin{aligned}&H_1=BC=\left( {\begin{array}{*{5}c} 0&{} u (1-{\mathcal S})\\ 1&{}u{\mathcal S}-{\mathcal S}^{-1}u \end{array}}\right) \left( {\begin{array}{*{5}c} (1-S^{-1})u&{}0\\ 0&{}1 \end{array}}\right) ;\\&H_2=AC=\left( {\begin{array}{*{5}c} u({\mathcal S}+1)&{}u(1-{\mathcal S})v\\ -v&{}0 \end{array}}\right) \left( {\begin{array}{*{5}c} (1-{\mathcal S}^{-1})u&{}0\\ 0&{}1 \end{array}}\right) , \end{aligned}$$

where *A* and *B* are of the total order 1 and their kernels are of dimension 1 Therefore both recursion operator $R=AB^{-1}$ and its inverse $R^{-1}=BA^{-1}$ are weakly nonlocal, and

$$\begin{aligned}&R=\left( {{\begin{array}{*{5}c} u{\mathcal S}+u+v_{1}+u_{1}+u{\mathcal S}^{-1} &{} u{\mathcal S}+u \\ v+v{\mathcal S}^{-1} &{} v \end{array}} } \right) -\left( {\begin{array}{*{5}c} u_t \\ v_t \end{array}}\right) ({\mathcal S}-1)^{-1} \left( {\begin{array}{*{5}c} \frac{1}{u}&0 \end{array}}\right) ;\\&R^{-1}= \left( {\begin{array}{*{5}c} \frac{1}{v_1} &{}-\frac{u}{v_1^2} {\mathcal S}+\frac{u}{v^2}-\frac{2u}{v v_1}\\ -{\mathcal S}^{-1} \frac{1}{v}-\frac{1}{v_1}&{} \frac{u}{v_1^2} {\mathcal S}+{\mathcal S}^{-1} \frac{u}{v^2} +\frac{2 u}{v v_1}+\frac{1}{v}\end{array}} \right) +\left( {\begin{array}{*{5}c} \frac{u}{v_{1}} -\frac{u}{v}\\ \frac{u_{-1}}{v_{-1}}-\frac{u}{v_1} \end{array}}\right) ({\mathcal S}-1)^{-1} \left( {\begin{array}{*{5}c} \frac{1}{u}&-\frac{2}{v} \end{array}}\right) . \end{aligned}$$

Note that the kernel of *A* is spanned by $$\left( {\begin{array}{*{5}c}0\\ \frac{1}{v}\end{array}} \right) $$, the kernel of $A^\dagger$ is spanned by $$\left( {\begin{array}{*{5}c} \frac{1}{u} \\ -\frac{2}{v} \end{array}} \right) $$ and $$B\left( {\begin{array}{*{5}c}0\\ \frac{1}{v}\end{array}} \right) =\left( {\begin{array}{*{5}c} \frac{u}{v}-\frac{u}{v_{1}} \\ \frac{u}{v_1}-\frac{u_{-1}}{v_{-1}}\end{array}} \right) $$. This explains the nonlocal term in the inverse of the recursion operator.

### The Ablowitz–Ladik lattice

Consider the Ablowitz–Ladik lattice [19]

$$\begin{aligned} \left\{ {{\begin{array}{*{5}c} u_t=(1-u v) (\alpha u_1-\beta u_{-1}) \\ v_t= (1- uv) (\beta v_1-\alpha v_{-1}). \end{array}} } \right. \end{aligned}$$

Its recursion operator [48]

62 $$\begin{aligned}&R=\left( {\begin{array}{*{5}c} (1-uv)~{\mathcal S}- u_1 v -u v_{-1} &{}-uu_{1} \\ vv_{-1}&{}(1-uv)~{\mathcal S}^{-1}\end{array}} \right) +\left( {\begin{array}{*{5}c} -u\\ v \end{array}}\right) ({\mathcal S}-1)^{-1} \left( {\begin{array}{*{5}c} v_{-1}&u_{1} \end{array}}\right) \nonumber \\&\quad -\left( {\begin{array}{*{5}c} (1-uv) u_{1}{} 1-uv) v_{-1} \end{array}}\right) ({\mathcal S}-1)^{-1} \left( {\begin{array}{*{5}c} \frac{v}{1-uv}&\frac{u}{1-uv} \end{array}}\right) \end{aligned}$$

can be written as $R=AB^{-1},$ where by letting $w=1-u v$, $w_i={\mathcal S}^i w$ and $p=u_1 v-u v_{-1}$ we have

$$\begin{aligned}&A=\!\left( \!{{\begin{array}{*{5}c} w {\mathcal S}\left( \frac{uv}{v_1}-u_1\right) +\frac{u_1vw}{v_1}-\frac{u v_{-1}w_1}{v_1} &{}w {\mathcal S}\frac{u}{p} (1-{\mathcal S}^{-1})-\frac{u^2 v_{-1}}{p}+\frac{u u_1 v}{p} {\mathcal S}^{-1} \\ w {\mathcal S}^{-1} (v_{-1}-\frac{v^2}{v_1})-u_1 v v_{-1}+\frac{u v^2 v_{-1}}{v_1}&{} -w{\mathcal S}^{-1}\frac{v}{p}(1-{\mathcal S}^{-1})+\frac{uv v_{-1}}{p}-\frac{u_1 v^2}{p}{\mathcal S}^{-1}\end{array}} } \!\right) \end{aligned}$$

and

$$\begin{aligned} B=\left( {{\begin{array}{*{5}c} \frac{w}{v}{\mathcal S}^{-1} p-u_1 w+\frac{uv w_1}{v_1}&{} \frac{u}{p}(1-{\mathcal S}^{-1})\\ v_{-1} w-\frac{v^2 w_1}{v_1}&{}-\frac{v}{p}(1-{\mathcal S}^{-1})\end{array}} } \right) . \end{aligned}$$

The operator *A* can be factorized as follows:

$$\begin{aligned}&\left( {{\begin{array}{*{5}c} 1 &{} 0\\ (v_{-2}v-v_{-1}^2)w r {\mathcal S}^{-1}&{} r\end{array}}} \right) \\&\quad \left( {\begin{array}{*{5}c} \frac{q (u_1vw-u v_{-1}w_1)}{(uv-u_1v_1)}&{} -\frac{w}{(v_{-1}v_1w_1-v^2w)} {\mathcal S}-\frac{u_1vw-u v_{-1}w_1}{(uv-u_1v_1)(v_{-2}vw-v_{-1}^2w_{-1})}\\ 0&{} 1 \end{array}} \right) D, \end{aligned}$$

whereNote that $\mathrm{Ord} A=\mathrm{Ord} D=2$ and $\ker D=\ker A$, which is spanned by

$$\begin{aligned} \mathbf{h}^{(1)}=\left( {\begin{array}{*{5}c} -\frac{1}{u_1 v-u v_{-1}}\\ \frac{v}{v_1} \end{array}}\right) \quad \text{ and }\quad \mathbf{h}^{(2)}=\left( {\begin{array}{*{5}c}\frac{u v_1}{u_1v-u v_{-1}}\\ u_1v_1-1 \end{array}}\right) . \end{aligned}$$

Thus the operator *A* is a full kernel operator and hence the inverse of $A B^{-1}$ is weakly nonlocal. Note that $\ker D^{\dagger }$ is spanned by

$$\begin{aligned} \mathbf{g}^{(1)}= & {} \left( {\begin{array}{*{5}c}vq\\ 0 \end{array}}\right) =\left( {\begin{array}{*{5}c}\frac{v}{(v_{-2}v w-v_{-1}^2 w_1)(v_{-1}v_1w_1-v^2w)}\\ 0 \end{array}}\right) \quad \text{ and }\\ \mathbf{g}^{(2)}= & {} \left( {\begin{array}{*{5}c}-\frac{v_{-1}q}{w}\\ 0 \end{array}}\right) . \end{aligned}$$

Thus $\ker A^{\dagger }$ is spanned by

$$\begin{aligned}&\left( {\begin{array}{*{5}c} 1 &{} {\mathcal S}w(v_{-1}^2-v_{-2}v)\\ 0 &{} \frac{1}{r} \end{array}} \right) \left( {\begin{array}{*{5}c} \frac{(uv-u_1v_1)}{q (u_1vw-u v_{-1}w_1)}&{}0\\ {\mathcal S}^{-1}\frac{w(uv-u_1v_1)(v_{-2}vw-v_{-1}^2w_{-1})}{u_1v w-u v_{-1}w_1}+v_{-1}v_1w_1-v^2w &{}1\end{array}}\right) \mathbf{g}^{(1)}\\&\quad =\left( {\begin{array}{*{5}c} v_1\\ u_{-1}\end{array}}\right) \end{aligned}$$

and similarly $$\left( {\begin{array}{*{5}c} \frac{v}{1-uv} \\ \frac{u}{1-uv}\end{array}}\right) $$. Moreover, we have

$$\begin{aligned} B(\mathbf{h}^{(1)})=\left( {\begin{array}{*{5}c}u\\ -v\end{array}}\right) ; \qquad B(\mathbf{h}^{(2)})=\left( {\begin{array}{*{5}c} (1-uv)u_{-1}\\ -(1-uv) v_{1} \end{array}}\right) . \end{aligned}$$

These give us the nonlocal term appearing in the inverse operator as stated in Theorem 1, and indeed

$$\begin{aligned}&R^{-1} =\left( {\begin{array}{*{5}c} (1-uv)~{\mathcal S}^{-1}&{}uu_{-1} \\ -vv_{1}&{} (1-uv)~{\mathcal S}-uv_1-u_{-1}v \end{array}} \right) \\&\quad +\left( {\begin{array}{*{5}c} u\\ -v \end{array}}\right) ({\mathcal S}-1)^{-1} \left( {\begin{array}{*{5}c} v_1&u_{-1} \end{array}}\right) \\&\quad +\left( {\begin{array}{*{5}c} (1-uv) u_{-1}\\ -(1-uv) v_1 \end{array}}\right) ({\mathcal S}-1)^{-1} \left( {\begin{array}{*{5}c} \frac{v}{1-uv}&\frac{u}{1-uv} \end{array}}\right) . \end{aligned}$$

### The Kaup–Newell lattice

Consider the Kaup–Newell lattice [49]:

$$\begin{aligned} \left\{ {\begin{array}{*{5}c} u_t=a\left( \frac{u_1}{1-u_{1}v_{1}}-\frac{u}{1-uv}\right) +b\left( \frac{u}{1+uv_{1}}-\frac{ u_{-1}}{1+u_{-1}v}\right) \\ v_t=a\left( \frac{v}{1-u v}-\frac{v_{-1}}{1-u_{-1} v_{-1}}\right) +b\left( \frac{v_1}{1+uv_{1}}-\frac{v}{1+u_{-1}v}\right) \end{array}} \right. :=a K_1 +b K_{-1}. \end{aligned}$$

Its recursion operator

$$\begin{aligned} R= & {} \left( {{\begin{array}{*{5}c} -\frac{1}{(1-u_1v_1)^2} {\mathcal S}+\frac{1}{(1-uv)^2}-\frac{2 u_1 v}{(1-u_1v_1)(1-uv)} &{}-\frac{u_1^2}{(1-u_1 v_1)^2} {\mathcal S}+\frac{u^2}{(1-u v)^2}-\frac{2 u u_1}{(1-uv)(1-u_1 v_1)} \\ -\frac{v_{-1}^2}{(1-u_{-1} v_{-1})^2} {\mathcal S}^{-1}-\frac{v^2}{(1-u v)^2}&{} -\frac{1}{(1-u_{-1}v_{-1})^2} {\mathcal S}^{-1}+\frac{1-2uv}{(1-uv)^2}\end{array}}}\right) \\&-2 K_1 ({\mathcal S}-1)^{-1} \left( {\begin{array}{*{5}c} \frac{v}{1-u v}&\frac{u}{1-u v}\end{array}}\right) , \end{aligned}$$

can be written as $R=AB^{-1},$ where

$$\begin{aligned}&A=\left( {{\begin{array}{*{5}c} ({\mathcal S}-1)\frac{1}{v(1-uv)}(1-{\mathcal S}^{-1})+2 ({\mathcal S}-1)\frac{u}{1-uv}{\mathcal S}^{-1} &{}({\mathcal S}-1)\frac{u}{1-uv} \\ (1-{\mathcal S}^{-1})\frac{v}{1-uv}({\mathcal S}^{-1}+1)&{} (1-{\mathcal S}^{-1})\frac{v}{1-uv}\end{array}}}\right) \\&\quad = \left( {{\begin{array}{*{5}c} {\mathcal S}-1 &{} 0\\ 0&{}1-{\mathcal S}^{-1}\end{array}}} \right) \left( {\begin{array}{*{5}c} \frac{u}{1-uv}&{}0\\ 0&{}\frac{v}{1-uv} \end{array}}\right) \left( {\begin{array}{*{5}c} 2- \frac{1}{uv}&{}1\\ 1&{}1 \end{array}}\right) \left( {\begin{array}{*{5}c} {\mathcal S}^{-1}-1&{}0\\ 2&{}1 \end{array}}\right) \end{aligned}$$

and

$$\begin{aligned} B=\left( {{\begin{array}{*{5}c} \frac{1-u v}{v}({\mathcal S}^{-1}-1)&{}-u \\ 0&{}v\end{array}} } \right) . \end{aligned}$$

The operator *A* does not have a full kernel since $\mathrm{Ord} A=3$ and its kernel is spanned by $$\left( {\begin{array}{*{5}c} 1\\ -2 \end{array}} \right) $$. Surprisingly, operator $C=A-B$ can be factorised as follows:

$$\begin{aligned} \left( {\begin{array}{*{5}c} 1&{} \frac{1}{v_1^2}{\mathcal S}\\ 0 &{}1 \end{array}} \right) \left( {\begin{array}{*{5}c} 1&{}0\\ (uv-{\mathcal S}^{-1}) \frac{v_1^2}{1-u^2 v_1^2} &{}1\end{array}}\right) \left( {\begin{array}{*{5}c} \frac{1+u v_1}{v_1^2(1-uv)}&{} 0\\ 0&{} 1 \end{array}} \right) D, \end{aligned}$$

where

$$\begin{aligned} D=\left( {\begin{array}{*{5}c} (v-2v_1+uv v_1)+v(1-u v_1) {\mathcal S}^{-1}&{} v(1-u^2 v_1^2) \\ v(1-{\mathcal S}^{-1})\frac{1+u v_1}{1-u v_1} &{} 0 \end{array}} \right) . \end{aligned}$$

Note that $\mathrm{Ord} C=\mathrm{Ord} D=1$ and $\ker D=\ker C$, which is spanned by $$\mathbf{h}=\left( {\begin{array}{*{5}c}\frac{1-u v_1}{1+uv_1}\\ \frac{2v_1}{v(1+u v_1)}-\frac{2}{(1+u_{-1}v)} \end{array}}\right) $$. Thus operator *C* is a full kernel operator and hence the inverse of $(A-B) B^{-1}$ is weakly nonlocal as presented in [20] and it equals to

63 $$\begin{aligned} (R-\mathrm {id})^{-1}= & {} {} \left( \!\! {{\begin{array}{*{5}c} \frac{1}{(1+u_{-1}v)^2} {\mathcal S}^{-1} -\frac{1+2 u v_1}{(1+u v_1)^2} &{}{} -\frac{u^2}{(1+u v_1)^2} {\mathcal S}+\frac{u_{-1}^2}{(1+u_{-1} v)^2}-\frac{2 u u_{-1}}{(1+u_{-1}v) (1+u v_1)}\\ \ \ -\frac{v^2}{(1+u_{-1} v)^2} {\mathcal S}^{-1}-\frac{v_1^2}{(1+u v_1)^2} &{}{} \frac{1}{(1+u v_1)^2} {\mathcal S}-\frac{1}{(1+u_{-1} v)^2}-\frac{2 u_{-1} v_1}{(1+u_{-1} v) (1+u v_1)} \end{array}} } \right) \nonumber \\&-2 K_{-1} ({\mathcal S}-1)^{-1} \left( {\begin{array}{*{5}c} \frac{v_1}{1+u v_1}&\frac{u_{-1}}{1+u_{-1} v}\end{array}}\right) . \end{aligned}$$

Note that $\ker D^{\dagger }$ is spanned by $$\left( {\begin{array}{*{5}c}0\\ \frac{1}{v} \end{array}}\right) $$ and thus $\ker C^{\dagger }$ is spanned by

$$\begin{aligned} \left( {\begin{array}{*{5}c} 1&{} 0\\ cS^{-1} \frac{1}{v_1^2} &{}1 \end{array}} \right) \left( {\begin{array}{*{5}c} 1&{}-\frac{v_1^2}{1-u^2 v_1^2} (uv-{\mathcal S})\\ 0 &{}1\end{array}}\right) \left( {\begin{array}{*{5}c} \frac{v_1^2(1-uv)}{1+u v_1}&{} 0\\ 0&{} 1 \end{array}} \right) \left( {\begin{array}{*{5}c}0\\ \frac{1}{v} \end{array}}\right) =\left( {\begin{array}{*{5}c}\frac{v_1}{1+u v_1}\\ \frac{u_{-1}}{1+u_{-1}v} \end{array}}\right) .\end{aligned}$$

Moreover, we have

$$\begin{aligned} B(\mathbf{h})=2 \left( {\begin{array}{*{5}c}\frac{u}{1+u v_1}-\frac{u_{-1}}{1+u_{-1}v}\\ \frac{v_1}{1+u v_1}-\frac{v}{1+u_{-1}v} \end{array}}\right) =2 K_{-1}. \end{aligned}$$

These give us the nonlocal term appearing in the inverse operator as shown in (63).

## Conclusions

In this paper we have built a rigorous algebraic setting for difference and rational (pseudo–difference) operators with coefficients in a difference field ${\mathrm F}$ and study their properties. In particular, we formulate a criteria for a rational operator to be weakly nonlocal. We have defined and studied preHamiltonian pairs, which is a generalization of the well known bi-Hamiltonian structures in the theory of integrable systems. By definition a preHamiltonian operator is an operator whose images form a Lie subalgebra in the Lie algebra of evolutionary derivations of ${\mathrm F}$. The latter can be directly verified and it is a relatively simple problem comparing to the verification of the Jacobi identity for Hamiltonian operators. We have shown that a recursion Nijenhuis operator is a ratio of difference operators from a preHamiltonian pair. Thus for a given rational operator, to test whether it is Nijenhuis or not can be done systematically. We applied our theoretical results to integrable differential difference equations in two aspects:

- We have constructed a rational recursion operator *R* (53) for Adler–Postnikov integrable Eq. (43) and shown that it can be written as the ratio of a preHamiltonian pair and thus it is Nijenhuis. Moreover, we proved that *R* produces infinitely many commuting local symmetries;
- For a given recursion operator we can answer the question whether the inverse operator is weakly nonlocal and, if so, how to bring it to the standard weakly nonlocal from (examples in Section 6).

In Sect. 6.4 we show that for a weakly nonlocal recursion operator *R* which does not have a weakly nonlocal inverse, may exist a constant $\gamma \in {\mathrm k}$ such that $(R-\gamma \ \mathrm {id})^{-1}$ is weakly nonlocal. In other words, the total order of the difference operator $A-\gamma B$ in the factorisation $R=AB^{-1}$ may be lower for a certain choice of $\gamma$. This observation requires further investigation.

The concept of preHamiltonian operators deserves further attention. These operators naturally appear in the description of the invariant evolutions of curvature flows in homogeneous spaces in both continuous [50] and discrete [35] setting. In the future, we’ll look into the geometric implication of such operators.

In this paper, we mainly explored the relation between PreHamiltonian operators and Nijenhuis operators. We are going to investigate how preHamiltonian pairs relate to biHamiltonian pairs. In our forthcoming paper [34], we’ll present the following main result: *if His a Hamiltonian (a priori nonlocal, i.e. rational) operator, then to find a second HamiltonianKcompatible withHis the same as to find a preHamiltonian pairAandBsuch that*$AB^{-1}H$*is skew-symmetric*.

We have discovered that Adler–Postnikov integrable equation (43) is indeed a Hamiltonian system. This equation can be written as $u_t=H \delta _u (\ln u)$, where *H* is the following skew-symmetric rational operator

$$\begin{aligned} \begin{aligned} H&=u^2u_1u_2^2{\mathcal S}^2-{\mathcal S}^{-2}u^2u_1u_2^2+{\mathcal S}^{-1}uu_1(u+u_1)-uu_1(u+u_1){\mathcal S}\\&+u(1-{\mathcal S}^{-1})(1-uu_1)({{\mathcal S}}u-u{\mathcal S}^{-1})^{-1}(1-uu_1)({\mathcal S}-1)u. \end{aligned} \end{aligned}$$

In [34], we are going to show that *H* is a Hamiltonian operator for Eq. (43) and explain how it is related to the recursion operator (53).

## Appendix A. Basic Concepts for a Unital Associative Principal Ideal Ring

Recall the definitions of some basic concepts for a unital associative ring $\mathfrak {R}$ (see for example [25]).

A *left* (respectively *right*) *ideal* of $\mathfrak {R}$ is an additive subgroup $\mathfrak {I}\subset \mathfrak {R}$ such that $\mathfrak {R} \mathfrak {I}=\mathfrak {I}$ (resp. $\mathfrak {I}\mathfrak {R}=\mathfrak {I}$).

A left (resp. right) *principal* ideals generated by $a\in \mathfrak {R}$ is, by definition, $\mathfrak {R} a$ (resp. $a\mathfrak {R}$).

A ring is called a *principal ideal ring*, if every left and right ideal of the ring is principal. In what follows we assume that the ring $\mathfrak {R}$ is both a left and a right principal ideal ring, meaning that every left ideal of $\mathfrak {R}$ and every right ideal of $\mathfrak {R}$ is principal.

Given an element $$a\in \mathfrak {R}$$, an element *d* is called a *right* (resp. *left*) *divisor* of *a* if $$a=b d$$ (resp. $$a=db $$) for some $$b\in \mathfrak {R}$$. An element $$m\in \mathfrak {R}$$ is called *left* (resp. *right*) *multiple* of *a* if $$m=ba$$ (resp. $$m=ab$$) for some $$b\in \mathfrak {R}$$.

Given elements $a, b \in \mathfrak {R}$, their *right* (resp. *left*) *greatest common divisor* (*gcd*) is the generator *d* of the *left* (resp. *right*) ideal generated by *a* and *b*: $\mathfrak {R} a + \mathfrak {R} b = \mathfrak {R} d$ (resp. $a \mathfrak {R} + b\mathfrak {R} = d\mathfrak {R}$). It is uniquely defined up to multiplication by an invertible element. It follows that *d* is a right (resp. left) divisor of both *a* and *b*, and we have the *Bezout identity*$d = ua + vb$ (resp. $d = au + bv$) for some $u, v \in \mathfrak {R}$.

Similarly, the *left* (resp. *right*) *least common multiple* (*lcm*) of *a* and *b* is an element $m \in \mathfrak {R}$ defined uniquely, up to multiplication by an invertible element, as the generator of the intersection of the *left* (resp. *right*) principal ideals generated by *a* and by *b*: $\mathfrak {R} m = \mathfrak {R} a \cap \mathfrak {R} b$ (resp. $m\mathfrak {R} = a\mathfrak {R} \cap b\mathfrak {R}$).

We say that *a* and *b* are *right* (resp. *left*) *coprime* if their *right* (resp. *left*) greatest common divisor is 1 (or invertible), namely if the left (resp. right ) ideal that they generate is the whole ring $\mathfrak {R} a + \mathfrak {R} b =\mathfrak {R}$ (resp. $a\mathfrak {R} + b\mathfrak {R} = \mathfrak {R}$). In particular there exist $u,v\in \mathfrak {R}$ such that $ua+vb=1$ (resp. $au + bv = 1$).

An element $a\in \mathfrak {R}{\setminus } \{0\}$ is called a *right zero divisor* if there exists $b\in \mathfrak {R}{\setminus } \{0\}$ (called *a left zero divisor*) such that $ba=0$.

A non-zero element $a\in \mathfrak {R}$ is called *regular* if it is neither a left nor a right zero divisor. A set of regular elements $\mathfrak {R}^\times =\{a\in \mathfrak {R}\,|\, a \ \text{ is } \text{ regular }\}$ is a multiplicative monoid of $\mathfrak {R}$.

A ring $\mathfrak {R}$ is called a *domain*, if it does not have zero divisors.

A domain $\mathfrak {R}$ is called *right (left) Euclidean*, if there exists a function

$$\begin{aligned} \mathrm{Ord}:\, \mathfrak {R}{\setminus } \{0\}\mapsto {\mathbb {Z}}_{\ge 0}, \end{aligned}$$

such that

1. $$ \mathrm{Ord}\, (a)\le \mathrm{Ord}\, (ab)\ge \mathrm{Ord}\, (b),\quad \forall a,b\in \mathfrak {R}{\setminus } \{0\}$$,
2. for any $a,b\in \mathfrak {R},\ b\ne 0$ there exist unique $c_r,q_r\in \mathfrak {R}$ (resp. $c_l,q_l\in \mathfrak {R}$), such that
  $$\begin{aligned} a=b c_r+q_r=c_l b+q_l \end{aligned}$$
  and $q_r=0$ or $\mathrm{Ord}\, q_r< \mathrm{Ord}\, b$ (resp. $q_l=0$ or $\mathrm{Ord}\, q_l< \mathrm{Ord}\, b$).

A principal ideal ring $\mathfrak {R}$ satisfies the right (and left) Ore property (Theorem 2.2 (c) in [25]). Namely, for any $a\in \mathfrak {R},\ b\in \mathfrak {R}^\times$ there exist $c\in \mathfrak {R}^\times , d\in \mathfrak {R}$ (resp. $c_1\in \mathfrak {R}^\times , d_1\in \mathfrak {R}$) such that $ac=bd$ (resp. $c_1 a=d_1 b$).

### Lemma 5

Let $\mathfrak {R}$ be a principal ideal ring. Let *a* and *b* be two right coprime elements in $\mathfrak {R}$ with *b* regular. Then there exists two left coprime elements $c,d\in \mathfrak {R}$ with *c* regular such that $ca=db$. Moreover,

(i) if $cp=dq$ for some $p,q \in \mathfrak {R}$ then there exists $z \in \mathfrak {R}$ such that $p=az$ and $q=bz$;
(ii) if $pa=qb$ for some $p,q \in \mathfrak {R}$ then there exists $z \in \mathfrak {R}$ such that $p=zc$ and $q=zd$.

### Proof

It follows from the left Ore property that for $a,b\in \mathfrak {R}, \ b$ regular, there exist $c,d\in \mathfrak {R},\ c$ regular, such that $ca=db$. We can assume that *c* and *d* are left coprime. Otherwise, one can simplify on the left by their left greatest common divisor, which is regular since *c* is.

(i) Let $\mathfrak {I}=\{x \in \mathfrak {R} | \exists y \in \mathfrak {R}, dx=cy \}$. $\mathfrak {I}$ is a right ideal in $\mathfrak {R}$, hence it can be written as $h \mathfrak {R}$ for some $h \in \mathfrak {R}$. Obviously $b\in \mathfrak {I}$, thus there exists $g \in \mathfrak {R}$ such that $b=hg$ and both *g*, *h* are regular. Element *h* itself lies in $\mathfrak {I}$, therefore there exists $f \in \mathfrak {R}$ such that $dh=cf$. Multiplying the latter on the right by *g* we have $cfg=dhg=db=ca$, which implies that $fg=a$ since *c* is regular. Recall that *a* and *b* are right coprime. Therefore the equalities $a=fg$ and $b=hg$ imply that *g* is invertible in $\mathfrak {R}$.

Now let us assume that $cp=dq$ for some elements $p,q \in \mathfrak {R}$. By definition of $\mathfrak {I}$ there exists $w \in \mathfrak {R}$ such that $q=hw$. We can rewrite *q* as $q=hg g^{-1}w=b z$ where $z =g^{-1}w \in \mathfrak {R}$. Finally we note that $cp=dq=dbz=caz$ which implies $p=a z$ since *c* is regular.

Taking the left ideal $\mathfrak {J}=\{x \in \mathfrak {R} | \exists y \in \mathfrak {R}, xb=ya \}$ we prove part (ii) of the Lemma in a similar way. $\quad \square$

## Appendix B. Lemmas Used for the Proof of Proposition 13

We denote by $\pi$ the projection from the space of Laurent difference polynomials $\mathrm {A}$ to the space of difference polynomials $\mathrm {K}$ defined by letting $\pi (b)$ being the nonsingular part of *b* for all difference Laurent monomial $b \in \mathrm {A}$. For example,

$$\begin{aligned} \pi \left( u+\frac{uu_1}{u_2}\right) =u. \end{aligned}$$

If $L=\sum _{n \le N}{l_n S^n}$ is a Laurent series with coefficients being Laurent difference polynomials, we denote by $\pi (L)$ the series $\sum _{n \le N}{\pi (l_n) S^n}$.

### Lemma 6

Let $a,b,c,d \in \mathrm {K}$ and $n \in {\mathbb {Z}}$. Then $\pi [(a+bS^{-1})\Delta ^{-1}(c+dS^{-1})]$ is a difference operator.

### Proof

We have

64 $$\begin{aligned} \begin{aligned} (a+bS^{-1})\Delta ^{-1}(c+dS^{-1})&= \sum _{n \ge 1}{(ac_{-2n-1} \beta ^n+bd_{-2n}(\beta ^{n-1})_{-1})S^{-2n-1}}\\&\quad +\sum _{n \ge 0}{(ad_{-2n-1} \beta ^n+bc_{-2n-2}(\beta ^n)_{-1})S^{-2n-2}}\\&\quad +ac_{-1}\beta ^0 S^{-1}. \end{aligned} \end{aligned}$$

It is clear that for *n* large enough $\pi (ac_{-2n-1} \beta ^n)=0$ and similarly $\pi (bd_{-2n}(\beta ^{n-1})_{-1})=0$.

$$\begin{aligned} \pi \left( ac_{-2n-1} \beta ^n+bd_{-2n}(\beta ^{n-1})_{-1}\right) =0 \text { for } n \gg 0. \end{aligned}$$

Similarly,

$$\begin{aligned} \pi \left( ad_{-2n-1} \beta ^n+bc_{-2n-2}(\beta ^n)_{-1}\right) =0 \text { for } n \gg 0. \end{aligned}$$

$$\square $$

### Lemma 7

Let $a,b,c,d, \in \mathrm {K}$ and $e \in \mathrm {A}$. Then $\pi [(a+bS^{-1})\Delta ^{-1}eS^{-1} \Delta ^{-1}(c+dS^{-1})]$ is a difference operator.

### Proof

Let us expand $L=(a+bS^{-1})\Delta ^{-1}eS^{-1} \Delta ^{-1}(c+dS^{-1})$ as a Laurent series in $S^{-1}$:

65 $$\begin{aligned} L=&\left( a+bS^{-1}\right) \left( \sum _{n \ge 0}{\beta ^ne_{-2n-1} S^{-2n-1}}\right) \left( \sum _{k \ge 0}{\left( \beta ^k\right) _{-1}S^{-2k-1}}\right) \left( c_{-1}S^{-1}+d_{-1}S^{-2}\right) \nonumber \\ =&\left( a+bS^{-1}\right) \left( \sum _{m \ge 0}{\beta ^{m+1}\left( \sum _{n= 0}^m{\left( \frac{e}{u}\right) _{-2n-1}}\right) S^{-2m-2}}\right) \left( c_{-1}S^{-1}+d_{-1}S^{-2}\right) \nonumber \\ =&\sum _{m \ge 0}{ac_{-2m-3}\beta ^{m+1}\left( \sum _{n= 0}^m{\left( \frac{e}{u}\right) _{-2n-1}}\right) S^{-2m-3}}\nonumber \\&\quad +\sum _{m \ge 0}{bd_{-2m-4}\left( \beta ^{m+1}\right) _{-1}\left( \sum _{n= 0}^m{\left( \frac{e}{u}\right) _{-2n-2}}\right) S^{-2m-5}} \nonumber \\&\quad +\sum _{m \ge 0}{bc_{-2m-4}\left( \beta ^{m+1}\right) _{-1}\left( \sum _{n= 0}^m{\left( \frac{e}{u}\right) _{-2n-2}}\right) S^{-2m-4}}\nonumber \\&\quad +\sum _{m \ge 0}{ad_{-2m-3}\beta ^{m+1}\left( \sum _{n= 0}^m{\left( \frac{e}{u}\right) _{-2n-1}}\right) S^{-2m-4}}. \end{aligned}$$

After applying $\pi$ to the coefficients of this Laurent series expansion of *L*, we get a difference operator. Let us show it for the first summand in the last line of (65), namely that

$$\begin{aligned} \sum _{m \ge 0}{\pi \left( ac_{-2m-3}\beta ^{m+1}\left( \sum _{n= 0}^m{\left( \frac{e}{u}\right) _{-2n-1}}\right) \right) S^{-2m-3}} \end{aligned}$$

is a difference operator (the same argument applies to the remaining three summands). This follows from the claim that for large enough *m*, and for all $0 \le n \le m$,

66 $$\begin{aligned} \pi \left( ac_{-2m-3}\beta ^{m+1}\left( \frac{e}{u}\right) _{-2n-1}\right) =0. \end{aligned}$$

Indeed, if *e* can be written as a sum of Laurent monomials for which the degree of the numerators, as polynomials in the $u_i$’s are bounded by $m_e$, and if $m_a$ and $m_c$ denote the degrees of *a* and *c* as polynomials in the $u_i$’s, then (66) holds for $m>m_a+m_c+m_e$. $\quad \square$

### Lemma 8

Let *f* be a difference polynomial such that *R* is recursion for the equation $u_t=f$. Then there exists a difference polynomial *k* such that $f=u(k_2-k)$.

### Proof

Operator *R* given by (53) is recursion for $u_t=f$ which implies that $\ln (u)$ is a conserved density of *f*, or in other words that there is a difference polynomial *g* such that $f=u(g_1-g)$.

To conclude we need to prove that $g_1-g=k_2-k$ for some difference polynomial *k*, which is equivalent to say that $g=k_1+k+\rho$ for some constant $\rho$. We claim that this is the same as saying that

67 $$\begin{aligned} \sum _n{(-1)^n S^{-n}\left( \frac{\partial g}{\partial u_n}\right) }=0. \end{aligned}$$

Indeed, it is clear that $\sum _n{(-1)^n S^{-n}\frac{\partial }{\partial u_n}(S+1)}=0$ by (3) and that any constant satisfies (67). Conversely, if a difference polynomial *g* of order (*M*, *N*) satisfies (67), then there exists a difference polynomial *k* and a constant $\rho$ such that $g=k_1+k+\rho$. To check this, we proceed by induction on the total order of *g*. If it is zero, meaning that *g* is a function of $u_N$ for a single *N*, then *g* must be a constant. If not, say if *g* has order (*M*, *N*) with $M<N$, then $\frac{\partial g}{\partial u_N}$ does not depend on $u_M$. Consequently, we can write *g* as a sum $h+k$ where *k* has order $(M',N)$ with $M<M'$ and *h* has order $(M, N')$ with $N'<N$. Since *g* and $k+k_{-1}$ both satisfy (67), it follows that $h-k_{-1}$ must satisfy (67) as well, i.e. we reduced the problem to a difference polynomial of lesser total order.

The difference polynomial (67) is the remainder of the division of $g_*$ by $(S+1)$ on the left. Let us call it *r*:

68 $$\begin{aligned} g_*=(S+1)X+r, \quad r=\sum _n{(-1)^n S^{-n}\left( \frac{\partial g}{\partial u_n}\right) }, \end{aligned}$$

where *X* is some difference operator. We want to prove that $r=0$. It is equivalent to prove that the remainder $r'$ of the division of $g_* u (S^2-1)$ by $S+1$ on the left is 0. Indeed $r'=ur-(ur)_{-2}$ and *r* is a difference polynomial, therefore $r=0 \iff r'=0$.

We are going to deduce that $r'=0$ from the fact that *R* is recursion for $f=u(g_1-g)$. Note that $f_*=u(S-1)g_*+g_1-g$. Recall Eq. (54) where *R* was expressed as $(Q\Delta ^{-1}C+P)(S^2-1)^{-1}\frac{1}{u}$. By Definition (36) of a recursion operator we have

69 $$\begin{aligned} \begin{aligned}&(Q_*[f]-f_* Q)\Delta ^{-1}C-Q\Delta ^{-1}\Delta _*[f] \Delta ^{-1}C\\&\quad +Q\Delta ^{-1}C_*[f] +P(S+1)^{-1}g_* u (S^2-1)\\&\quad -f_* P+ P_*[f]+Q \Delta ^{-1}C (S+1)^{-1} g_* u (S^2-1)=0. \end{aligned} \end{aligned}$$

The idea is to expand (69) as a Laurent series in $S^{-1}$ and to project the coefficients in front of $S^{-N}$ for large *N* on the space of difference polynomials. Let us start by rearranging (69) using two Euclidean divisions

70 $$\begin{aligned} C= w_2+w_{-1}+Z(S+1),\qquad g_* u (S^2-1)=r'+(S+1)Y, \end{aligned}$$

where *Y* and *Z* are two difference operators. Combining (69) with (70), we get:

71 $$\begin{aligned} \begin{aligned}&Q \Delta ^{-1}(w_2+w_{-1})S^2(S+1)^{-1}r'+p(S+1)^{-1}r'\\&\quad =Q\Delta ^{-1}\Delta _*[f] \Delta ^{-1}C-(Q_*[f]-f_* Q)\Delta ^{-1}C\\&\qquad -Q\Delta ^{-1}(C_*[f]+CY+Zr')-Pr' +f_* P- P_*[f]. \end{aligned} \end{aligned}$$

By Lemmas 6 and 7, if *M* is the RHS of (71), $\pi (M)$ is a difference operator. Therefore,

72 $$\begin{aligned} \pi [ Q \Delta ^{-1}(w_2+w_{-1})S^2(S+1)^{-1}r'+p(S+1)^{-1}r'] \end{aligned}$$

must be a difference operator as well. Let us write $Q=a+bS^{-1}$ where $a=u(uu_1-1)$ and $b=u(1-uu_{-1})$ and let $c=w_2+w_{-1}$. Looking only at even powers of $S^{-1}$ in the Laurent series expansion of (72) we obtain

73 $$\begin{aligned}&\pi [(a(\beta ^0c_{-1}+\cdots +\beta ^Nc_{-2N-1})-b(\beta ^0c_{-1}+\cdots \nonumber \\&\quad +\beta ^{N-1}c_{ -2N+1})_{-1}-p){r'}_{-2N}]=0 \text { for all } N \gg 0, \end{aligned}$$

where the Laurent difference polynomials $\beta ^n=\frac{u_{-1}\ldots u_{-2n-1}}{u\ldots u_{-2n}}, n \ge 1$, $\beta ^0=\frac{1}{u}$ satisfy

74 $$\begin{aligned} \Delta ^{-1}=\sum _{n \ge 0}{\beta ^nS^{-2n-1}}. \end{aligned}$$

It is clear that for all $k> 1$ and for all $N \ge \mathrm{Ord}\, r' +2$, we have

$$\begin{aligned} \pi (ac_{2k-1}\beta ^k {r'}_{-2N})=\pi (b(c_{2k-1}\beta ^k)_{-1}{r'}_{-2N})=0. \end{aligned}$$

In other words, there exists $K \ge 0$ such that

75 $$\begin{aligned}&\pi [(a(\beta ^0c_{-1}+\cdots +\beta ^Kc_{-2K-1})-b(\beta ^0c_{-1}+\cdots \nonumber \\&\qquad +\beta ^{K-1}c_{ -2K+1})_{-1}-p){r'}_{-2N}]=0 \text { for all } N \gg 0. \end{aligned}$$

If $r' \ne 0$, $r'$ is either a constant or the order of ${r'}_{-2N}$ must go to $(-\infty ,-\infty )$ as *N* grows. In both cases we must have:

76 $$\begin{aligned} \pi [a(\beta ^0c_{-1}+\cdots +\beta ^Kc_{-2K-1})-b(\beta ^0c_{-1}+\cdots +\beta ^{K-1}c_{ -2K+1})_{-1}-p]=0. \end{aligned}$$

This quantity can be computed directly, and we obtain

77 $$\begin{aligned} \begin{aligned} p&=-2+u(u_2+3u_1+2u+u_{-1}+u_{-2})+2u_{-1}u_{-3} \\&-u(2u_1u_{-1}u_{-3}+2u_1uu_{-1}+uu_1u_2+u_{-2}u_{-1}u+2u_{-4}u_{-2}u) \end{aligned}, \end{aligned}$$

which is a contradiction to *p* given in (57). Thus we have $r'=0$ and hence $g=k_1+k+\rho$. By now we have proved the statement. $\quad \square$

### Lemma 9

Let $g \in \mathrm {K}$ be such that *R* is recursion for $f=u(g_2-g)$. Then

$$\begin{aligned} Q \Delta ^{-1} \left( C(g)_*u(S^2-1)-C(g)(S^2-S)\right) +\left( Q_*[f]-f_*Q-Q(g_1-g_2)\right) \Delta ^{-1}C \end{aligned}$$

is a difference operator.

### Proof

We have $\Delta ^{-1}_*[f]=(g_1-g_2)\Delta ^{-1}+\Delta ^{-1}(g_1-g_2)$ and $f_*=u(S^2-1)g_*+g_2-g$. From (69) we deduce that

78 $$\begin{aligned} Q \Delta ^{-1}\left( C g_*u(S^2-1)-(g_2-g_1)C+C_*[f]\right) +\left( Q_*[f]-f_*Q-Q(g_2-g_1)\right) \Delta ^{-1}C \end{aligned}$$

is a difference operator. It remains to rewrite the first nonlocal term. We have modulo left multiplication by $\Delta$ and we have

$$\begin{aligned} \begin{aligned} C g_*u(S^2-1)&=C(g)_*u(S^2-1)-(g_2(w_2)_*-g_1(w_{-1})_*)u(S^2-1) \\&=C(g)_*u(S^2-1)+u_1u_3g_2(S^5-S)-uu_{-2}g_1(S^2-S^{-2}) \\&\equiv C(g)_*u(S^2-1)+(uu_{-2}g_{-2}-u_1u_3g_2)S-u_1u_3(g_5-g_1)S^2 \end{aligned} \end{aligned}$$

and

$$\begin{aligned} C_*[f]=uu_{-2}(g_2-g_{-2})S-u_1u_3(g_5-g_1)S^2 . \end{aligned}$$

Therefore

79 $$\begin{aligned} C g_*u(S^2-1)-(g_2-g_1)C+C_*[f] \equiv C(g)_*u(S^2-1)-C(g)(S^2-S). \end{aligned}$$

We conclude combining (78) to (79). $\quad \square$

### Lemma 10

Let *a*, *b*, *c*, *d*, *e*, *f*, *g*, *h* be difference Laurent polynomials such that $a, b, g, h \ne 0$ and

$$\begin{aligned} (a+bS^{-1})\Delta ^{-1}(c+dS^{-1})+(e+fS^{-1})\Delta ^{-1}(g+hS^{-1}) \end{aligned}$$

is a difference operator. Then there exists a constant $\lambda \in {\mathrm k}$ such that

$$\begin{aligned} \begin{aligned} e+fS^{-1}&=\lambda (a+bS^{-1})\\ c+dS^{-1}&=-\lambda (g+hS^{-1}). \end{aligned} \end{aligned}$$

### Proof

Recall the definition of the Laurent monomials $\beta ^n$ for $n \ge 0$

80 $$\begin{aligned} \Delta ^{-1}=\sum _{n \ge 0}{\beta ^n S^{-2n-1}}. \end{aligned}$$

We have

81 $$\begin{aligned} \begin{aligned} (a+bS^{-1})\Delta ^{-1}(c+dS^{-1})&= \sum _{n \ge 1}{(ac_{-2n-1} \beta ^n+bd_{-2n}(\beta ^{n-1})_{-1})S^{-2n-1}}\\&\quad +\sum _{n \ge 0}{(ad_{-2n-1} \beta ^n+bc_{-2n-2}(\beta ^n)_{-1})S^{-2n-2}}\\&\quad +ac_{-1}\beta ^0 S^{-1}. \end{aligned} \end{aligned}$$

Therefore, we must have for large enough *n*

$$\begin{aligned} \begin{aligned} \beta ^{n-1}(ad_{-2n+1}+eh_{-2n+1})+(\beta ^{n-1})_{-1}(bc_{-2n}+fg_{-2n})&=0 \\ \beta ^{n}(ac_{-2n-1}+eg_{-2n-1})+(\beta ^{n-1})_{-1}(bd_{-2n}+fh_{-2n})&=0. \end{aligned} \end{aligned}$$

Here $\beta ^n$ has poles at $u,u_{-2},\ldots ,u_{-2n}$ ($\beta ^n=\frac{u_{-1}\ldots u_{-2n+1}}{u\ldots u_{-2n}}$) and $(\beta ^{n-1})_{-1}$ has poles at $u_{-1},\ldots ,u_{-2n+1}$. Moreover, the Laurent polynomials inside the parenthesis can only have a bounded number of poles, independently of *n*. Combining these two facts we deduce that for large *n* the arguments inside the four parenthesis must vanish:

82 $$\begin{aligned} \begin{aligned} 0&=ad_{-2n+1}+eh_{-2n+1} \\ 0&=bc_{-2n}+fg_{-2n}\\ 0&=ac_{-2n-1}+eg_{-2n-1}\\ 0&=bd_{-2n}+fh_{-2n}, \quad \quad n \gg 0. \end{aligned} \end{aligned}$$

Since $a,b,g,h \ne 0$, either $e=f=c=d=0$, in which case we can take $\lambda =0$, or $e,f,c,d, \ne 0$. In the latter case we conclude using the fact that, if two Laurent difference polynomials *x* and *y* are such that $x_{2n}=y$ for infinitely many $n \in {\mathbb {Z}}$, then *x* and *y* are both equal to the same constant. $\quad \square$

### Lemma 11

Let *d* be a difference polynomial. Then *d* is in the image of $\Delta$ if and only if

83 $$\begin{aligned} \mathrm{d}_* u (1+S^{-1})-d=\Delta P, \end{aligned}$$

where *P* is a difference operator. In this case, we have

84 $$\begin{aligned} d=\Delta \left( -S\left( \sum _{n}{\frac{\alpha _{2n}}{u}\left( \frac{\partial d}{\partial u_{2n}}\right) _{-2n}}\right) \right) . \end{aligned}$$

Here for all $n \in {\mathbb {Z}}$, $\alpha _{2n}$ (resp. $\alpha _{2n+1}$) is the unique difference Laurent polynomial such that $S^{2n}u-\alpha _{2n}$ (resp. $S^{2n+1}u-\alpha _{2n+1}S^{-1}$) is divisible on the left by $\Delta$. Moreover,

$$\begin{aligned} \sum _{n}{\frac{\alpha _{2n}}{u}\left( \frac{\partial d}{\partial u_{2n}}\right) _{-2n}} \end{aligned}$$

is a difference polynomial.

### Proof

Suppose that $d=u_1d'_1-ud'_{-1}$ for a difference Laurent polynomial $d'$. Then the Fréchet derivative of *d* expands as:

$$\begin{aligned} \mathrm{d}_*=\Delta \mathrm{d}'_*+ d'_1S-d'_{-1}. \end{aligned}$$

Hence (we use to $\equiv$ to denote modulo left multiplication by $\Delta$) we get

$$\begin{aligned} \mathrm{d}_*u(1+S^{-1}) \equiv (Sd'-d'_{-1})u(1+S^{-1}) \equiv u_1d'_1-ud'_{-1} \equiv d. \end{aligned}$$

Conversely assume that

85 $$\begin{aligned} \mathrm{d}_* u (1+S^{-1}) \equiv d. \end{aligned}$$

Recall that the $\alpha _n$’s are defined so that $S^{2n}u \equiv \alpha _{2n}$ and $S^{2n+1}u \equiv \alpha _{2n+1} S^{-1}$ for all $n \in {\mathbb {Z}}$. The following identity can be easily checked by induction

86 $$\begin{aligned} \alpha _{2n+2}=\frac{u}{u_{-1}} \alpha _{2n+1}=\frac{u^2}{u_{-1}u_{-2}}(\alpha _{2n})_{-2}, \quad \forall n \in {\mathbb {Z}}. \end{aligned}$$

Let us rewrite the LHS of (85):

87 $$\begin{aligned} \begin{aligned}&\sum _{n}{S^n\left( \frac{\partial d}{\partial u_n}\right) _{-n}u(1+S^{-1})}\\&\quad =\sum _{n}{S^n u\left( \left( \frac{\partial d}{\partial u_n}\right) _{-n}+\frac{u_1}{u}\left( \frac{\partial d}{\partial u_{n+1}}\right) _{-n}\right) }\\&\quad \equiv \sum _{n}{\alpha _{2n}\left( \left( \frac{\partial d}{\partial u_{2n}}\right) _{-2n}+ \frac{u_1}{u} \left( \frac{\partial d}{\partial u_{2n+1}}\right) _{-2n}\right) }\\&\qquad +\sum _{n}{\alpha _{2n+1}S^{-1}\left( \left( \frac{\partial d}{\partial u_{2n+1}}\right) _{-2n-1}+ \frac{u_1}{u} \left( \frac{\partial d}{\partial u_{2n+2}}\right) _{-2n-1}\right) }. \end{aligned} \end{aligned}$$

Combining (85), (86) and (87) we obtain

88 $$\begin{aligned} \begin{aligned} d&= \sum _{n}{\alpha _{2n}\left( \frac{\partial d}{\partial u_{2n}}\right) _{-2n}}+\frac{u_1}{u}\sum _{n}{\alpha _{2n}\left( \frac{\partial d}{\partial u_{2n+1}}\right) _{-2n}}, \\ 0&=\sum _{n}{\frac{u}{u_{-2}}(\alpha _{2n})_{-2}\left( \frac{\partial d}{\partial u_{2n+1}}\right) _{-2n-2}}+\sum _{n}{\alpha _{2n+2}\left( \frac{\partial d}{\partial u_{2n+2}}\right) _{-2n-2}} \end{aligned} \end{aligned}$$

from which it follows that

$$\begin{aligned} \begin{aligned} d&=\Delta \left( -\sum _{n}{\frac{(\alpha _{2n})_{-1}}{u_{-1}}\left( \frac{\partial d}{\partial u_{2n+1}}\right) _{-2n-1}}\right) \\&=\Delta \left( -S\left( \sum _{n}{\frac{\alpha _{2n}}{u}\left( \frac{\partial d}{\partial u_{2n}}\right) _{-2n}}\right) \right) . \end{aligned} \end{aligned}$$

We proved that there exists a Laurent difference polynomial $d'$ such that $d=u_1d'_1-ud'_{-1}$. It implies that $d'$ cannot have poles (since its highest pole should be lesser or equal than 0 and its lowest pole should be greater than 0), therefore that it is a difference polynomial. $\quad \square$
